# On a variant of Pillai’s problem involving *S*-units and Fibonacci numbers

**DOI:** 10.1007/s40590-022-00450-7

**Published:** 2022-07-15

**Authors:** Volker Ziegler

**Affiliations:** University of Salzburg, Hellbrunnerstrasse 34/I, 5020 Salzburg, Austria

**Keywords:** Baker’s method, Fibonacci numbers, Diophantine equations, 11D61, 11B39, 11D45, 11Y50

## Abstract

Let us denote by $F_n$ the *n*-th Fibonacci number. In this paper we show that there exist at most finitely many integers *c* such that the exponential Diophantine equation $F_n-2^x3^y=c$ has more than one solution $(n,x,y)\in {\mathbb {N}}^3$ with $n>1$. Moreover, in the case that $c>0$ we find all integers *c* such that the Diophantine equation has at least three solutions and in the case that $c<0$ we find all integers *c* such that the Diophantine equation has at least four solutions.

## Introduction

A famous conjecture due to Pillai [18] states that the Diophantine equation

1 $$\begin{aligned} a^x-b^y=c \end{aligned}$$

has for fixed integer $c>0$ at most finitely many solutions (*a*, *b*, *x*, *y*) in positive integers.

In the case that *a*, *b* and *c* are fixed Pillai showed that for *c* larger than some constant which may depend on *a* and *b* Diophantine equation (1) has at most one solution (*x*, *y*). In particular, Stroeker and Tijdeman [20] showed that for $a=3$ and $b=2$ at most one solution exists provided that $c>13$.

In more recent years several researchers considered variants of Pillai’s problem involving recurrence sequences. Ddamulira, Luca and Rakotomalala [10] considered the Diophantine equation

$$\begin{aligned} F_n -2^m = c. \end{aligned}$$

Here and in the rest of the paper $F_n$ denotes the *n*-th Fibonacci number which is defined by $F_0=0$, $F_1=1$ and $F_{n+2}=F_{n+1}+F_n$ for $n\ge 0$. Instead of Fibonacci numbers also other recurrence sequences were considered. In particular, if we consider the equation

$$\begin{aligned} U_n-V_m=c \end{aligned}$$

various results can be found in the literature, where the authors proved that for *c* large enough the Diophantine equation has at most one solution (see Table 1, where $F_n$ are Fibonacci numbers, $F_n^{(k)}$ are *k*-generalized Fibonacci numbers, $T_n$ are Tribonacci numbers, $P\!e_n$ are Pell numbers and $P\!a_n$ are Padovan numbers). Let us also note that the case for general recurrence sequences has been considered by Chim, Pink and the author [5].

**Table 1:** List of results on variants of Pillai’s problem

$U_n$	$V_m$	References
$F_n$	$2^m$	Ddamulira, Luca and Rakotomalala [10]
$T_n$	$2^m$	Bravo, Luca and Yazán [2]
$F_n$	$T_m$	Chim, Pink and Ziegler [4]
$F^{(k)}_n$	$2^m$	Ddamulira, Gómez and Luca [8]
$T_n$	$3^m$	Ddamulira [7]
$P\!a_n$	$3^m$	Ddamulira [6]
$F_n$	$P\!e_m$	Hernández, Luca and Rivera [14]
$F^{(k)}_n$	$3^m$	Ddamulira and Luca [9]

In this paper we consider the following Diophantine equation:

$$\begin{aligned} F_n-2^x3^y=c, \end{aligned}$$

where *x*, *y* and *n* are integers with $x,y\ge 0$ and $n\ge 2$. In particular, we are interested in the number of solutions. First, note that, if $(n,x,y)=(1,x,y)$ is a solution, then (2, *x*, *y*) is also a solution to $F_n-2^x3^y=c$, since $F_1=F_2=1$. This is the reason why we exclude solutions with $n\le 1$.

Our first observation is the following theorem:

### Theorem 1

There exists a finite set ${\mathcal {C}}$ such that the Diophantine equation

2 $$\begin{aligned} F_n-2^x3^y=c, \qquad n\ge 2 \end{aligned}$$

has at least two solutions $(n,x,y)\in {\mathbb {N}}^3$, if and only if $c\in {\mathcal {C}}$.

Unfortunately our proof depends on ineffective methods, so we cannot determine the set ${\mathcal {C}}$ in Theorem 1. In order to obtain an effective version of Theorem 1 we have to assume the existence of a third solution in the case that $c>0$. In this case we obtain the following theorem:

### Theorem 2

Let $c>0$ be an integer such that the Diophantine equation (2) has at least three solutions $(n,x,y)\in {\mathbb {N}}^3$, then $c\in {\mathcal {C}}_3$, with

$$\begin{aligned} {\mathcal {C}}_3=\{1,2,4,5,7,17,53,215\} \end{aligned}$$

and Equation (2) has for no $c>0$ more than five solutions.

In particular, in the case that $c\in {\mathcal {C}}_3$ all solutions to (2) are listed in Table 2.

Our proof of Theorem 2 cannot be generalized to the case that $c<0$. However, assuming the existence of a fourth solution we can circumvent problems that appear by generalizing the proof of Theorem 2 to the case that $c<0$ and we obtain:

### Theorem 3

Let $c<0$ be an integer such that the Diophantine equation (2) has at least four solutions $(n,x,y)\in {\mathbb {N}}^3$, then $c\in {\mathcal {C}}_4$, with

$$\begin{aligned} {\mathcal {C}}_4=\{-1,-3,-7,-11,-19\} \end{aligned}$$

and Equation (2) has for no $c<0$ more than five solutions.

In particular, in the case that $c\in {\mathcal {C}}_4$ all solutions to (2) are listed in Table 2.

**Table 2:** List of solutions $(n_1,x_1,y_1),\dots , (n_5,x_5,y_5)$ to (2) with $c\in {\mathcal {C}}_3 \cup {\mathcal {C}}_4\cup \{0\}$ and $n_1\le \cdots \le n_5$

*c*	$(n_1,x_1,y_1)$	$(n_2,x_2,y_2)$	$(n_3,x_3,y_3)$	$(n_4,x_4,y_4)$	$(n_5,x_5,y_5)$
$-19$	(5, 3, 1)	(6, 0, 3)	(7, 5, 0)	(11, 2, 3)	
$-11$	(2, 2, 1)	(5, 4, 0)	(7, 3, 1)	(8, 5, 0)	
$-7$	(2, 3, 0)	(3, 0, 2)	(5, 2, 1)	(11, 5, 1)	(14, 7, 1)
$-3$	(2, 2, 0)	(4, 1, 1)	(5, 3, 0)	(7, 4, 0)	(8, 3, 1)
$-1$	(2, 1, 0)	(3, 0, 1)	(4, 2, 0)	(5, 1, 1)	(6, 0, 2)
0	(2, 0, 0)	(3, 1, 0)	(4, 0, 1)	(6, 3, 0)	(12, 4, 2)
1	(3, 0, 0)	(4, 1, 0)	(5, 2, 0)	(7, 2, 1)	(10, 1, 3)
2	(4, 0, 0)	(5, 0, 1)	(6, 1, 1)	(9, 5, 0)	
4	(5, 0, 0)	(6, 2, 0)	(7, 0, 2)		
5	(6, 0, 1)	(7, 3, 0)	(8, 4, 0)		
7	(6, 0, 0)	(7, 1, 1)	(9, 0, 3)	(10, 4, 1)	
17	(8, 2, 0)	(11, 3, 2)	(13, 3, 3)		
53	(10, 1, 0)	(11, 2, 2)	(14, 2, 4)		
215	(13, 1, 2)	(14, 1, 4)	(22, 3, 7)		

Let us note that Theorems 2 and 3 together with Table 2 and Lemma 9, which resolves the case that $c=0$, yield the following corollary:

### Corollary 1

The Diophantine equation $F_n-2^x3^y=c$ with $(n,x,y)\in {\mathbb {N}}^3$ and $n>1$ has for a fixed integer *c* at most five solutions.

Finally we would like to have a good guess what the set ${\mathcal {C}}$ of Theorem 1 actually is. Therefore we prove the following theorem:

### Theorem 4

Let $|c|\le 10^{100}$ be an integer such that the Diophantine equation (2) has at least two solutions $(n,x,y)\in {\mathbb {N}}^3$, then $c\in {\mathcal {C}}_s$, with

$$\begin{aligned} {\mathcal {C}}_s&=\{-209719, -7768, -4606, -4574, -2291, -2215, -2043, -1694, -1118,\\&\quad -1063, -1011, -1003, -969, -919, -775, -721, -647, -640, -542, -504, -487, \\&\quad -478,-431, -427, -343, -288, -254, -253, -240, -235, -222, -199, -190, -161,\\&\quad -158, -154, -141, -131, -127, -123, -107, -103, -95, -94, -91, -73, -62, -59,\\&\quad -55, -53, -51, -47, -46, -43, -41, -38, -35, -33, -31, -30, -27, -26, -24, -23,\\&\quad -22, -19, -17, -16, -15, -14, -13, -11, -10, -9, -8, -7, -6, -5, -4, -3, -2, -1,\\&\quad 0, 1, 2, 3, 4, 5, 7, 9, 10, 12, 15, 16, 17, 18, 19, 25, 28, 31, 41, 53, 71, 80, 85, 89, 161, 185,\\&\quad 215, 578, 933, 1581, 1589, 4173, 9794, 28081, 28369, 74737\}. \end{aligned}$$

For a complete list of all solutions to (2) with $c\in {\mathcal {C}}_s$ see the “Appendix”, Table 3.

In view of the results in Theorem 4 the following conjecture seems plausible.

### Conjecture 1

We have

$$\begin{aligned} {\mathcal {C}}={\mathcal {C}}_s. \end{aligned}$$

## Outline of the paper

Let us assume that Diophantine equation (2) has two solutions (*n*, *x*, *y*) and $(n_1,x_1,y_1)$ with $n,n_1>1$. Note that $n=n_1$ implies $2^x3^y=2^{x_1}3^{y_1}$ and hence $x=x_1$, $y=y_1$. Thus we may assume that $n>n_1$. First let us note that a solution $(n,n_1,x,x_1,y,y_1)\in {\mathbb {N}}^6$ to

3 $$\begin{aligned} F_n-F_{n_1}=2^x3^y-2^{x_1}3^{y_1}, \end{aligned}$$

with $n>n_1>1$ implies that for $c=F_n-2^x3^y$ the Diophantine Eq. (2) has at least two solutions. That is for a complete resolution of conjecture 1 we have to completely solve Diophantine Eq. (3).

Let us note that Eq. (3) is a *S*-unit equation and by the theory of *S*-unit equations we can prove Theorem 1 in Sect. 4.

In Sect. 5 we consider the Diophantine inequality

$$\begin{aligned} |F_n-2^x3^y|\le 10^{100} \end{aligned}$$

and show by standard techniques that all solutions (*n*, *x*, *y*) to this inequality satisfy $n\le 525$, which allows us to compute a complete list of solutions to this inequality. Searching for solutions that yield the same value

$$\begin{aligned} c=F_n-2^x3^y \end{aligned}$$

yields a proof of Theorem 4.

For a solution $(n,n_1,x,x_1,y,y_1)$ to (3) we write $X=x\log 2+y\log 3$. With this notation we will prove in Sect. 6 that $n\log \alpha = X+O((\log n)^2)$ (explicit constants are computed in Sect. 6). This result together with Theorem 4 yields that if the Diophantine Eq. (2) has at least two solutions (*n*, *x*, *y*) and $(n_1,x_1,y_1)$ with $n,n_1>1$ for a $c\not \in {\mathcal {C}}_s$, then we may assume that $n>400$.

The heart of the paper are the proofs of Theorems 2 and 3. The proofs are based on lower bounds for linear forms in complex and *p*-adic logarithms. In Sect. 7 we prove under the assumptions that $c>0$ and that a third solution to (2) that there exists a huge but absolute bound for *n*. In the case that $c<0$ we prove under the assumption that a fourth solution exists a huge but absolute bound for *n*. In the Sects. 9 and 10 we use LLL-reduction methods, the theory of continued fractions and *p*-adic reduction methods due to Pethő and de Weger [17] to find a rather small bound for *n*, which will conclude the proofs of the Theorems 2 and 3.

In the next Section we provide several helpful Lemmas. In particular, we discuss several tools form Diophantine number theory, e.g., lower bounds for linear forms in logarithms and LLL-reduction via approximation lattices.

## Auxiliary results

First we start with collecting some facts on Fibonacci numbers. Let us start with the Binet-formula which is well known:

$$\begin{aligned} F_n=\frac{\alpha ^n-\beta ^n}{\sqrt{5}}, \quad \text {with} \quad \alpha =\frac{1+\sqrt{5}}{2}, \;\; \beta =\frac{1-\sqrt{5}}{2}. \end{aligned}$$

Let us note that $\beta =-\alpha ^{-1}$. Assume that $n\ge 10$ then we get

4 $$\begin{aligned} 0.447 \alpha ^n< \frac{\alpha ^n}{\sqrt{5}}\cdot (1 - \alpha ^{-20}) \le F_n\le \frac{\alpha ^n}{\sqrt{5}}\cdot (1 + \alpha ^{-20}) < 0.448 \alpha ^n. \end{aligned}$$

Let us note that in the case that $c>0$ we have

$$\begin{aligned} 2^x3^y=F_n-c<F_n-1<\alpha ^n. \end{aligned}$$

Taking logarithms this yields

5 $$\begin{aligned} x<n\frac{\log \alpha }{\log 2}\quad \text {and}\quad y<n\frac{\log \alpha }{\log 3}. \end{aligned}$$

In the case that $c<0$ we have

$$\begin{aligned} F_n=2^x3^y+c<2^x3^y \end{aligned}$$

and taking logarithms yields in view of inequality (4) the following inequality

6 $$\begin{aligned} n \log \alpha <2 \max \{x \log 2,y\log 3\}. \end{aligned}$$

Furthermore let us recall a simple fact form calculus.

### Lemma 1

If $x\in {\mathbb {R}}$ satisfies $|x|<1/2$, then $|\log (1+x)|<\frac{3}{2} |x|$.

In order to obtain absolute upper bounds we will apply results on lower bounds for linear forms in logarithms. To state these results we need the notion of height. Therefore let $\alpha \ne 0$ be an algebraic number of degree *d* and let

$$\begin{aligned} a_0(X-\alpha _1)\cdots (X-\alpha _d) \in {\mathbb {Z}}[X] \end{aligned}$$

be the minimal polynomial of $\alpha$. Then the absolute logarithmic Weil height is defined by

$$\begin{aligned} h(\alpha )=\frac{1}{d} \left( \log |a_0|+\sum _{i=1}^d \max \{0,\log |\alpha _i|\}\right) . \end{aligned}$$

With this basic notation we have the following result on lower bounds for linear forms in logarithms due to Matveev [16].

### Lemma 2

(Matveev 2000) Denote by $\alpha _1,\dots ,\alpha _n$ algebraic numbers, $\ne 0,1$, by $\log \alpha _1,\dots ,\log \alpha _n$ determinations of their logarithms, by *D* the degree over ${\mathbb {Q}}$ of the number field $K={\mathbb {Q}}(\alpha _1,\dots ,\alpha _n)$, and by $b_1,\dots , b_n$ rational integers. Furthermore let $\kappa = 1$ if *K* is real and $\kappa = 2$ otherwise. For all integers *j* with $1\le j \le n$ choose

$$\begin{aligned} A_j\ge h'(\alpha _j)=\max \{Dh(\alpha _j),|\log \alpha _j|,0.16\}, \end{aligned}$$

and set

$$\begin{aligned} B= \max \left\{ \{1\}\cup \{|b_j|A_j/A_n\, :\, 1\le j \le n\}\right\} . \end{aligned}$$

Assume that

$$\begin{aligned} \Lambda =b_1\log \alpha _1 +\cdots +b_n\log \alpha _n\ne 0. \end{aligned}$$

Then

$$\begin{aligned} \log |\Lambda |\ge - C(n,\kappa ) \max \{1,n/6\}C_0 W_0 D^2\Omega \end{aligned}$$

with

$$\begin{aligned}&\Omega =A_1\cdots A_n\\&C(n,\kappa )=\frac{16}{n! \kappa } e^n (2n+ 1 + 2\kappa ) (n+ 2)(4(n+ 1))^{n+1}\left( \frac{en}{2} \right) ^\kappa ,\\&C_0= \log (e^{4.4n+7}n^{5.5}D^2\log (eD)),\quad W_0= \log (1.5eBD\log (eD)) \end{aligned}$$

In most of our applications we will be in the situation, where $K={\mathbb {Q}}(\sqrt{5})$ and $n=4$. In this case Matveev’s lower bound is

$$\begin{aligned} \log |\Lambda |> - 3.45\cdot 10^{13} \Omega \log (13.81 B). \end{aligned}$$

Also note that instead of *B* one can use $B^*=\max _{1\le j \le n}\{|b_j|\}$ in Matveev’s bound.

However, in the case that the number of logarithms is two we have numerically rather good results due to Laurent [15]. In particular, we will use the following result:

### Lemma 3

(Laurent 2008) Let $\alpha _1, \alpha _2 \ge 1$ be two multiplicatively independent real algebraic numbers, let $b_1$ and $b_2$ be two positive integers and let

$$\begin{aligned} \Lambda := b_2 \log \alpha _2 - b_1 \log \alpha _1. \end{aligned}$$

Then

$$\begin{aligned} \log |\Lambda | \ge - 17.9 D^4 (\max \{ \log b' + 0.38, 30/D, 1\})^2 \log A_1 \log A_2, \end{aligned}$$

where

$$\begin{aligned} D&=[{\mathbb {Q}}(\alpha _1,\alpha _2):{\mathbb {Q}}],\\ b'&=\frac{b_1}{D \log A_2} + \frac{b_2}{D \log A_1},\\ \log A_i&\ge \max \{ h(\alpha _i),|\log \alpha _i|/D,1/D\} \qquad (i=1,2). \end{aligned}$$

We also apply a *p*-adic variant of Laurent’s result due to Bugeaud and Laurent [3, Corollary 1]. Before we can state their result we need some more notations. For a prime *p* we denote by ${\mathbb {Q}}_p$ the field of *p*-adic numbers with the standard *p*-adic valuation $v_p$. As above let $\alpha _1,\alpha _2$ be algebraic numbers over ${\mathbb {Q}}$ and we regard them as elements of the field $K_p={\mathbb {Q}}_p(\alpha _1,\alpha _2)$ and we write $D=[K_p:{\mathbb {Q}}_p]$. Similar as in the case of Matveev’s theorem above we have to use a modified height. In particular, we write

$$\begin{aligned} h'(\alpha _i) \ge \max \left\{ h(\alpha _i),\log p/D \right\} , \ (i=1,2). \end{aligned}$$

### Lemma 4

(Bugeaud and Laurent 2008) Let $b_1,b_2$ be positive integers and suppose that $\alpha _1$ and $\alpha _2$ are multiplicatively independent algebraic numbers such that $v_p(\alpha _1)=v_p(\alpha _2)=0$. Put

$$\begin{aligned} E':=\frac{b_1}{h'(\alpha _2)}+\frac{b_2}{h'(\alpha _1)}. \end{aligned}$$

and

$$\begin{aligned} E:=\max \left\{ \log E'+\log \log p+0.4,10,10\log p\right\} . \end{aligned}$$

Then we have

$$\begin{aligned} v_p(\alpha _1^{b_1}\alpha _2^{b_2}-1)\le \frac{24 pg}{(p-1)(\log p)^4} E^2D^4h'(\alpha _1)h'(\alpha _2), \end{aligned}$$

where $g>0$ is the smallest integer such that $v_p(\alpha _i^g-1)>0$.

In order to apply these results on lower bounds for linear forms in logarithms we have to compute the height of several quantities. In particular we have

$$\begin{aligned}&h(2)=\log 2,\quad h(3)=\log 3,\\&h(\sqrt{5})=\frac{1}{2} \left( \log \left| \sqrt{5}\right| +\log \left| -\sqrt{5}\right| \right) =\log \sqrt{5} \end{aligned}$$

and

$$\begin{aligned} h(\alpha )=h(\beta )=\frac{1}{2}\left( \max \{0,\log |\alpha |\} +\max \{0,\log |\beta |\}\right) =\frac{\log \alpha }{2}. \end{aligned}$$

Let us also state some standard properties of the height, where $\eta ,\gamma \in \bar{{\mathbb {Q}}}$ and $\ell \in {\mathbb {Q}}$:

$$\begin{aligned} h(\eta \pm \gamma )&\le h(\eta ) + h(\gamma ) + \log 2, \\ h(\eta \gamma ^{\pm 1})&\le h(\eta ) + h(\gamma ),\\ h(\eta ^\ell )&= |\ell | h(\eta ). \end{aligned}$$

With these properties we can easily compute:

7 $$\begin{aligned} h(\alpha ^x\pm 1)=h(\beta ^x\pm 1)\le \frac{|x| \log \alpha }{2}+\log 2. \end{aligned}$$

In order to apply Lemma 4 we have to ensure that $\alpha _1$ and $\alpha _2$ are multiplicatively independent. In our application of Lemma 4 we have to ensure that $\alpha$ and $\tau (t)=\frac{\alpha ^t-1}{\beta ^t-1}$ are multiplicatively independent. Therefore the following lemma will be helpful:

### Lemma 5

Let $t\ge 1$ be an integer. The algebraic numbers $\alpha$ and $\tau (t)$ are multiplicatively dependent if and only if $t=1,3$ or *t* is even. In particular, we have $\tau (1)=-\alpha ^{-2}$, $\tau (3)=-\alpha ^2$ and $\tau (2t)=-\alpha ^{2t}$.

### Proof

A quick computation shows

$$\begin{aligned} \tau (2t)=\frac{\alpha ^{2t}-1}{\beta ^{2t}-1}=\frac{\alpha ^{2t}-1}{\alpha ^{-2t}-1}=-\alpha ^{2t}. \end{aligned}$$

Therefore we may assume that *t* is odd, hence $\beta ^t=-\alpha ^{-t}$.

First, we note that $\alpha$ is a fundamental unit in $K={\mathbb {Q}}(\sqrt{5})$ and therefore $\alpha$ and $\tau (t)$ are multiplicatively dependent, if and only if $\tau (t)$ is a unit. It is also easy to see that $\tau (t)<-1$ for all $t>1$ and that $\tau (1)=-\alpha ^{-2}$. Thus we have to find all pairs $(x,t)\in {\mathbb {N}}^2$ with $t>1$ odd, such that $\tau (t)=-\alpha ^x$. Hence we have to solve the equation

$$\begin{aligned} \tau (t)=\frac{\alpha ^t-1}{\beta ^t-1}=\frac{\alpha ^t-1}{-\alpha ^{-t}-1}=-\alpha ^x. \end{aligned}$$

Some easy calculations show that the equation above implies

8 $$\begin{aligned} \alpha ^t=\alpha ^x+\alpha ^{x-t}+1. \end{aligned}$$

Since

$$\begin{aligned} \alpha ^x<\alpha ^t=\alpha ^x+\alpha ^{x-t}+1<2\alpha ^x<\alpha ^{x+2} \end{aligned}$$

we get $t=x+1$. Thus (8) turns into

$$\begin{aligned} \alpha ^{x+1}-\alpha ^x=\alpha ^{-1}+1. \end{aligned}$$

But we have $\alpha ^{-1}+1=\alpha$ and $\alpha ^{x+1}-\alpha ^{x}=\alpha ^{x-1}$ which yields $x=2$ and we get $\tau (3)=-\alpha ^2$. $\square$

### Remark 1

In the case of $\tilde{\tau }(t)=\frac{\alpha ^t+1}{\beta ^t+1}$ a similar result was proved in [22, Lemmas 3 and 4]. In particular, we have that $\tilde{\tau }(t)$ and $\alpha$ are multiplicatively dependent if and only if *t* is even or $t=1,3$. In the case of multiplicative dependence we have $\frac{\alpha ^{2t}+1}{\beta ^{2t}+1}=\alpha ^{2t}$, $\frac{\alpha ^{3}+1}{\beta ^{3}+1}=\alpha ^{4}$ and $\frac{\alpha +1}{\beta +1}=\alpha ^{4}$.

Lemma 5 shows that in infinitely many cases Lemma 4 is not applicable and we have to estimate $v_p(\alpha ^x\pm 1)$. This can be done by using the so-called *p*-adic logarithm $\log _p$ (e.g., see [19, Sect. II.2.4.] for a more detailed approach).

Let ${\mathbb {C}}_p$ be the complex *p*-adic field, which is complete with respect to $|\cdot |_p$ and is algebraic closed. Then there exists a function $\log _p(x)$, the so-called *p*-adic logarithm, defined on all ${\mathbb {C}}_p$ such that $\log _p(xy) = \log _p(x) + \log _p(y)$ and $\log _p(\zeta )=\log _p(p^s)=0$ for all roots of unity $\zeta$ and all $s\in {\mathbb {Z}}$. Moreover, for every $\xi \in {\mathbb {C}}_p$ with $|\xi - 1|_p < p^{-1/(p-1)}$ the *p*-adic logarithm is defined by

$$\begin{aligned} \log _p \xi = -\sum _{i=1}^{\infty } \frac{(1-\xi )^i}{i}. \end{aligned}$$

Moreover, we have

9 $$\begin{aligned} |\log _p \xi |_p = |\xi - 1|_p, \quad \text {respectively} \quad v_p (\log _p \xi ) = v_p(\xi -1) \end{aligned}$$

provided that $$|\xi - 1|_p < p^{-1/(p-1)}$$.

Using *p*-adic logarithms we can prove the following helpful lemma:

### Lemma 6

Let $x\ge 0$ be an integer, then we have

$$\begin{aligned} v_2(\alpha ^x-1)&=\left\{ \begin{array}{ll} 1+v_2(x) &{} \qquad \text {if}\;\;3| x\\ 0 &{} \qquad \text {if}\;\;3 \not \mid x \end{array}\right. \\ v_2(\alpha ^x+1)&=\left\{ \begin{array}{ll} 1 &{} \qquad \text {if}\;\;3| x\\ 0 &{} 3\qquad \text {if}\;\; \not \mid x \end{array}\right. \\ v_3(\alpha ^x-1)&=\left\{ \begin{array}{ll} 1+v_3(x) &{} \qquad \text {if}\;\;8| x\\ 0 &{} \qquad \text {if}\;\;8  \not \mid  x \end{array}\right. \\ v_3(\alpha ^x+1)&=\left\{ \begin{array}{ll} 1+v_3(x) &{} \qquad \text {if}\;\;x\equiv 4 \mod 8\\ 0 &{} \qquad \text {if}\;\;x\not \equiv 4 \mod 8 \end{array}\right. \end{aligned}$$

### Proof

We start with the case that $p=3$ and note that (3) is an inert prime ideal of ${\mathbb {Q}}(\sqrt{5})$. Hence, $\langle \alpha \rangle =\{\alpha ^x \mod 3\, : \, x\in {\mathbb {Z}}\}$ has order at most 8. By a simple computer search we find

$$\begin{aligned} \alpha ^x\left\{ \begin{array}{cc} \equiv 1 \mod 3 &{} \quad \text {if}\;\;x\equiv 0 \mod 8; \\ \not \equiv 1 \mod 3 &{} \quad \text {if}\;\;x\not \equiv 0 \mod 8, \end{array}\right. \end{aligned}$$

and

$$\begin{aligned} \alpha ^x\left\{ \begin{array}{cc} \equiv -1 \mod 3 &{} \quad \text {if}\;\;x\equiv 4 \mod 8; \\ \not \equiv -1 \mod 3 &{} \quad \text {if}\;\;x\not \equiv 4 \mod 8. \end{array}\right. \end{aligned}$$

Moreover, we easily find that $v_3(\alpha ^8-1)=1$ and $v_3(\alpha ^4+1)=1$, that is we have

$$\begin{aligned} |\alpha ^8-1|_3\le \frac{1}{3}<3^{-1/2} \end{aligned}$$

and

$$\begin{aligned} {|}(-\alpha ^4)-1|_3=|\alpha ^4+1|_3 =\le \frac{1}{3}<3^{-1/2}. \end{aligned}$$

Therefore we may apply (9) in the cases described below.

Assume for the moment that 8|*x*. Then we have

$$\begin{aligned} v_3(\alpha ^x-1)&=v_3(\log _3(\alpha ^x))=v_3\left( \frac{x}{8} \log _3(\alpha ^8)\right) \\&= v_3\left( \frac{x}{8}\right) +v_3\left( \log _3(\alpha ^8)\right) =v_3(x)+1. \end{aligned}$$

In the case that $x\equiv 4 \mod 8$ we obtain

$$\begin{aligned} v_3(\alpha ^x+1)&= v_3(-\alpha ^x-1)=v_3(\log _3(-\alpha ^x)) =v_3\left( \frac{x}{4} \log _3(\alpha ^4)\right) \\&= v_3\left( \frac{x}{4}\right) +v_3\left( \log _3(\alpha ^4)\right) =v_3(x)+1. \end{aligned}$$

Let us note that by similar computations as in the case that $p=3$ we can deduce the results for $p=2$. However, let us note that another proof of the case $p=2$ is given in [22, Corollary 1 and 2]. $\square$

Applications of lower bounds for linear forms in logarithms usually yield huge upper bounds, which make a direct brute force verification of the remaining cases unfeasible. However with approximation lattices and the so called LLL-reduction method we have a method to reduce these huge bounds.

Let ${\mathcal {L}}\subseteq {\mathbb {R}}^k$ be a *k*-dimensional lattice with LLL-reduced basis $b_1,\dots ,b_k$ and denote by *B* the matrix with columns $b_1,\dots , b_k$. Moreover, we denote by $b^*_1,\dots ,b^*_k$ the orthogonal basis of ${\mathbb {R}}^k$ which we obtain by applying the Gram-Schmidt process to the basis $b_1,\dots ,b_k$. In particular, we have that

$$\begin{aligned} b^*_i=b_i-\sum _{j=1}^{i-1}\mu _{i,j}b^*_j, \qquad \mu _{i,j} =\frac{\langle b_i,b_j\rangle }{\langle b_j^*,b_j^*\rangle }. \end{aligned}$$

Further, let us define

$$\begin{aligned} l({\mathcal {L}},y)={\left\{ \begin{array}{ll} \min _{x \in {\mathcal {L}}} \Vert x-y\Vert , &{} y \not \in {\mathcal {L}}, \\ \min _{0 \ne x \in {\mathcal {L}}} \Vert x\Vert , &{} y \in {\mathcal {L}}, \end{array}\right. } \end{aligned}$$

where $\Vert \cdot \Vert$ denotes the euclidean norm on ${\mathbb {R}}^k$. It is well known, that by applying the LLL-algorithm it is possible to give in polynomial time a lower bound for $l({\mathcal {L}},y) \ge c_1$ (e.g., see [19, Sect. V.4]):

### Lemma 7

Let $y\in {\mathbb {R}}^k$ and $z=B^{-1}y$ with $z=(z_1,\dots ,z_k)^T$. Furthermore we define:

- If $y\not \in {\mathcal {L}}$ let $i_0$ be the largest index such that $z_{i_0}\ne 0$ and put $\sigma =\{z_{i_0}\}$, where $\{\cdot \}$ denotes the distance to the nearest integer.
- If $y\in {\mathcal {L}}$ we put $\sigma =1$.

Finally let

$$\begin{aligned} c_2=\max _{1\le j\le k}\left\{ \frac{\Vert b_1\Vert ^2}{\Vert b_j^*\Vert ^2} \right\} . \end{aligned}$$

Then we have

$$\begin{aligned} l({\mathcal {L}},y)^2\ge c_2^{-1}\sigma ^2 \Vert b_1\Vert ^2=c_1^2. \end{aligned}$$

In our applications we suppose that we are given real numbers $\eta _0,\eta _1,\dots ,\eta _k$ linearly independent over ${\mathbb {Q}}$ and two positive constants $c_3, c_4$ such that

10 $$\begin{aligned} |\eta _0+x_1\eta _1+\cdots +x_k\eta _k| \le c_3\exp (- c_4H), \end{aligned}$$

where the integers $x_i$ are bounded by $|x_i| \le X_i$ with $X_i$ given upper bounds for $1 \le i \le k$. We write $X_0=\max _{1 \le i \le k}\{X_i\}$. The basic idea in such a situation, due to de Weger [11], is to approximate the linear form (10) by an approximation lattice. Namely, we consider the lattice ${\mathcal {L}}$ generated by the columns of the matrix

$$\begin{aligned} {\mathcal {A}}=\begin{pmatrix} 1 &{} 0 &{} \dots &{} 0 &{} 0 \\ 0 &{} 1 &{} \dots &{} 0 &{} 0 \\ \vdots &{} \vdots &{} \vdots &{} \vdots &{} \vdots \\ 0 &{} 0 &{} \dots &{} 1 &{} 0 \\ \lfloor { C\eta _1}\rfloor &{} \lfloor { C\eta _2}\rfloor &{} \dots &{} \lfloor { C\eta _{k-1}}\rfloor &{} \lfloor { C\eta _k}\rfloor \end{pmatrix} \end{aligned}$$

where *C* is a large constant usually of the size of about $X_0^k$. Let us assume that we have an LLL-reduced basis $b_1,\dots ,b_k$ of ${\mathcal {L}}$ and that we have a lower bound $l({\mathcal {L}},y)\ge c_1$ with $y=(0,0,\dots ,-\lfloor {C\eta _0}\rfloor )$. Note that $c_1$ can be computed by using the results of Lemma 7. Then we have with these notations the following lemma (e.g. see [19, Lemma VI.1]):

### Lemma 8

Let us write $S=\sum _{i=1}^{k-1}X_i^2$ and $T=\frac{1+\sum _{i=1}^k{X_i}}{2}$. If $c_1^2 \ge T^2+S$, then Inequality (10) implies that we have either $x_1=x_2=\dots =x_{k-1}=0$ and $x_k=-\frac{\lfloor {C\eta _0}\rfloor )}{\lfloor { C\eta _k}\rfloor )}$ or

11 $$\begin{aligned} H \le \frac{1}{ c_4}\left( \log (C c_3)-\log \left( \sqrt{ c_1^2-S}-T\right) \right) . \end{aligned}$$

## Ineffective methods

The purpose of this section is to prove Theorem 1. For technical reasons we treat the case $c=0$ separately. Indeed it is easy to see that the equation

$$\begin{aligned} F_n-2^x3^y=0 \end{aligned}$$

has several solutions. For instance one easily finds the solutions

$$\begin{aligned} (n,x,y)=(3,1,0),(4,0,1),(6,3,0),(12,4,2), \end{aligned}$$

thus $0\in {\mathcal {C}}$. To show that these are indeed the only solutions, we recall the notation of a primitive divisor for some arbitrary sequence $(U_n)_{n\in {\mathbb {N}}}$ first.

### Definition 1

A prime number *p* is called a primitive divisor of $U_n$ if $p|U_n$ but $p\not \mid U_m$ for all $0\le m<n$.

The famous primitive divisor theorem (in its simplest form) due to Bilu, Hanrot and Voutier [1] states that each member $U_n$ of a Lucas sequence has a primitive divisor if $n>30$. Since $F_3=2$ and $F_4=3$ the equation $F_n=2^x3^y$ cannot have a solution with $n>30$. Checking the finitely many remaining cases we get:

### Lemma 9

The Diophantine equation (2) has in the case that $c=0$ exactly five solutions, namely

$$\begin{aligned} (n,x,y)=(2,0,0),(3,1,0),(4,0,1),(6,3,0),(12,4,2). \end{aligned}$$

The following result due to Evertse et al. [12] on *S*-unit equations is well-known:

### Lemma 10

(Evertse et.al. 2002) Let *K* be an algebraically closed field of characteristic zero. Let $K^*$ be the multiplicative group of nonzero elements and $\Gamma$ be a subgroup of $(K^*)^N$ of finite rank *r*. Given $a_1,\dots ,a_N\in K^*$ we denote by $A(a_1,\dots ,a_N;\Gamma )$ the number of solutions $(x_1,\dots ,x_n)\in \Gamma$ of the equation

$$\begin{aligned} a_1x_1+\cdots +a_Nx_N=0 \end{aligned}$$

such that no proper subsum of $a_1x_1+\cdots +a_Nx_N$ vanishes. Then

$$\begin{aligned} A(a_1,\dots ,a_N;\Gamma )\le \exp \left( (6N)^{3N}(r+1)\right) . \end{aligned}$$

We apply Lemma 10 to (3). In particular, (3) can be rewritten as

12 $$\begin{aligned} \frac{1}{\sqrt{5}}\alpha ^n -\frac{1}{\sqrt{5}}\alpha ^{n_1} -\frac{1}{\sqrt{5}}\beta ^n +\frac{1}{\sqrt{5}}\beta ^{n_1} -2^x3^y-2^{x_1}3^{y_1}=0. \end{aligned}$$

That is $N=6$, $\Gamma =\langle \alpha ,2,3\rangle$ and therefore $r=3$. Thus an application of Lemma 10 yields $|{\mathcal {C}}|\le \exp (36^{18}\cdot 4)$, provided that no solution to (12) which implies a solution to (3) has a vanishing subsum.

Since $\alpha =-\beta ^{-1}$, 2 and 3 are multiplicative independent, and since we assume that $n>n_1>1$ and since a solution to (3) implies that $2^x3^y>2^{x_1}3^{y_1}$ we conclude that no two-term subsum vanishes.

Since $2^x3^y$ and $2^{x_1}3^{y_1}$ are algebraic integers, but all other terms in (12) are not, we conclude that a vanishing three-term subsum contains either $2^x3^y$ or $2^{x_1}3^{y_1}$. Thus we may assume without loss of generality that a vanishing three-term subsum contains $2^x3^y$.

Let us consider the case that the subsum

$$\begin{aligned} -2^x3^y+\frac{1}{\sqrt{5}}\alpha ^n+\frac{1}{\sqrt{5}}\beta ^{n_1} \end{aligned}$$

vanishes, i.e. that $2^x3^y=\frac{1}{\sqrt{5}}\alpha ^n+\frac{1}{\sqrt{5}}\beta ^{n_1}$. Let $\sigma \in {{\,\mathrm{Gal}\,}}({\mathbb {Q}}(\sqrt{5})/{\mathbb {Q}})$ be the non-trivial ${\mathbb {Q}}$-automorphism, that is $\sigma (\alpha )=\beta$ and $\sigma (\beta )=\alpha$. Then we obtain

$$\begin{aligned}&\frac{1}{\sqrt{5}}\alpha ^n-\frac{1}{\sqrt{5}}|\beta |<\frac{1}{\sqrt{5}} \alpha ^n+\frac{1}{\sqrt{5}}\beta ^{n_1}=2^x3^y\\&\quad =\sigma (2^x3^y)=\frac{1}{\sqrt{5}}\beta ^n+\frac{1}{\sqrt{5}} \alpha ^{n_1}<\frac{1}{\sqrt{5}}\alpha ^{n_1}+\frac{1}{\sqrt{5}}\beta ^2, \end{aligned}$$

which implies $\alpha ^n-\alpha ^{n_1}< \beta ^2-\beta$. But this yields the contradiction

$$\begin{aligned} 1=\alpha ^2-\alpha \le \alpha ^n-\alpha ^{n_1}< \beta ^2-\beta =1. \end{aligned}$$

The case that one of the subsums

$$\begin{aligned}&-2^x3^y-\frac{1}{\sqrt{5}}\alpha ^{n_1}-\frac{1}{\sqrt{5}}\beta ^{n}\\&-2^x3^y+\frac{1}{\sqrt{5}}\alpha ^{n}-\frac{1}{\sqrt{5}}\alpha ^{n_1}\\&-2^x3^y-\frac{1}{\sqrt{5}}\beta ^{n}+\frac{1}{\sqrt{5}}\beta ^{n_1} \end{aligned}$$

can be treated similarly. In the case that

$$\begin{aligned} -2^x3^y+\frac{1}{\sqrt{5}}\alpha ^n-\frac{1}{\sqrt{5}}\beta ^{n} \end{aligned}$$

vanishes we have $F_n=2^x3^y$ and we have $c=0$, which has been excluded. In the case that the subsum

$$\begin{aligned} -2^x3^y-\frac{1}{\sqrt{5}}\alpha ^{n_1}+\frac{1}{\sqrt{5}}\beta ^{n_1} \end{aligned}$$

vanishes we obtain $2^x3^y=-F_{n_1}<0$ which is again a contradiction. This concludes the proof of Theorem 1.

## Proof of Theorem 4

In order to prove Theorem 4 completely we consider the Diophantine inequality

13 $$\begin{aligned} |F_n-2^x3^y|\le 10^{100}. \end{aligned}$$

To find all solutions to (13) in the case that *n* is small can be done by a brute force search. Indeed by (5) with *n* also *x* and *y* are small. Therefore we assume that $n>520$ to avoid technical difficulties. In particular, assuming that $n>520$ yields $\frac{10^{100}}{F_n}<0.001$ and $\frac{10^{100}}{\alpha ^n}<0.001$.

We start with rewriting Inequality (13) and get

$$\begin{aligned} \left| \frac{2^x3^y\sqrt{5}}{\alpha ^n}-1 \right| \le \frac{10^{100} \sqrt{5}}{\alpha ^n}+\frac{|\beta |^n}{\alpha ^n}. \end{aligned}$$

Taking logarithms and an application of Lemma 1 yields

14 $$\begin{aligned} |\Lambda |=|x\log 2+y\log 3 -n \log \alpha +\log \sqrt{5}|<\frac{3.36 \cdot 10^{100}}{\alpha ^n}. \end{aligned}$$

Since 2, 3, 5 and $\alpha$ are multiplicatively independent we have $\Lambda \ne 0$. Moreover, by assuming that $n>520$ we have

$$\begin{aligned} 2^x3^y\le F_n+10^{100}<1.001 F_n \end{aligned}$$

and by taking logarithms we obtain

$$\begin{aligned} 1.001n>n+ 0.003> \max \left\{ x \frac{\log 2}{\log \alpha },y \frac{\log 3}{\log \alpha } \right\} . \end{aligned}$$

That is $B<1.001 n$ in Matveev’s theorem (Lemma 2) and we obtain

$$\begin{aligned} n\log \alpha - 231.5 < 8.123 \cdot 10^{13} \log (13.83 n), \end{aligned}$$

which yields $n<6.6\cdot 10^{15}$, $x<4.59\cdot 10^{15}$ and $y<2.9\cdot 10^{15}$.

We apply the LLL-reduction method to the approximation lattice

$$\begin{aligned} {\mathcal {A}}=\begin{pmatrix} 1 &{} 0 &{} 0 \\ 0 &{} 1 &{} 0 \\ \lfloor { C \log 2}\rfloor &{} \lfloor { C\log 3}\rfloor &{} \lfloor { C\log \alpha }\rfloor \end{pmatrix} \end{aligned}$$

with $C=10^{55}$ and choose $y=(0,0,-\lfloor C \log \sqrt{5}\rfloor )$. An application of Lemma 7 yields

$$\begin{aligned} l({\mathcal {L}},y)\ge c_1=2.9721333170\dots \cdot 10^{17}. \end{aligned}$$

If we choose $X_1=4.59\cdot 10^{15}$, $X_2=2.9\cdot 10^{15}$ and $X_3=6.6\cdot 10^{15}$, then with the notations of Lemma 8 we have $c_1^2\ge T^2+S$ and an application to inequality (14), i.e. we choose $c_3=3.36\cdot 10^{100}$ and $c_4=\log \alpha$, yields

$$\begin{aligned} n \le 660, \quad x\le 460, \quad y\le 290. \end{aligned}$$

Repeating the reduction process, but this time with $C=10^{10}$, $X_1=460$, $X_2=290$ and $X_3=660$ yields

$$\begin{aligned} n\le 525, \quad x\le 364, \quad y\le 230 . \end{aligned}$$

To find all $|c|\le 10^{100}$ for which (2) has at least two solutions we compute for each triple (*n*, *x*, *y*) with $n\le 525, x\le 364$ and $y\le 230$ the quantity $c=F_n-2^x3^y$ and only consider those quadruples (*c*, *n*, *x*, *y*) for which $|c|\le 10^{100}$ holds. This yields a list of almost 17 million solutions to (13). Sorting the list for *c* and discarding all solutions where consecutive quadruples do not have the same value for *c* we find all integers *c*, with $|c|\le 10^{100}$ such that Diophantine equation (2) has at least two solutions. All these integers *c* are listed in Theorem 4, which proves the theorem. Let us note that the computation takes only a few minutes on a typical desktop PC.

For a complete list of all solutions to (2) with $c\in {\mathcal {C}}_s$ we refer to Table 3 in the appendix.

## Bounds for solutions to *S*-unit equations

The purpose of this section is to prove the following Proposition:

### Proposition 1

Let $\Delta >10^{80}$ be a fixed integer and assume that

15 $$\begin{aligned} 2^x3^y-2^{x_1}3^{y_1}=\Delta , \end{aligned}$$

then we have $2^x3^y<\Delta (\log \Delta )^{60 \log \log \Delta }$.

Let us note that Proposition 1 is a special instance of a more general result due to Tijdeman [21]. Let us note that Tijdeman’s result [21] implies a bound of the form $\Delta (\log \Delta )^{A}$ for some explicit computable constant *A*. However, we will use Laurent’s result [15] on lower bounds for linear forms in two logarithms to obtain a small numerical value for *A*, but get an additional factor $\log \Delta$ in the exponent.

### Proof of Proposition 1

We write $u=2^x3^y$ and $v=2^{x_1}3^{y_1}$, then (15) can be written as $u-v=\Delta$. First, we note that

16 $$\begin{aligned} \max \{|x-x_1|,|y-y_1|\} \log 2\le x \log 2+y\log 3=\log u. \end{aligned}$$

Let us divide Equation (15) through *u*, then we obtain

$$\begin{aligned} \left| 2^{x_1-x}3^{y_1-y}-1\right| \le \frac{\Delta }{u}. \end{aligned}$$

In view of Proposition 1 we may assume that $u>2\Delta$ and by taking logarithms we obtain

17 $$\begin{aligned} |\Lambda |=\left| (x-x_1)\log 2+(y-y_1)\log 3\right| \le 3/2 \frac{\Delta }{u}. \end{aligned}$$

Since $\log 2$ and $\log 3$ are linearly independent over ${\mathbb {Q}}$ the linear form $\Lambda$ only vanishes if $x=x_1$ and $y=y_1$ which contradicts our assumption that $\Delta \ne 0$.

Thus we apply Lemma 3 with $D=1$, $\log A_1=1$ and $\log A_2=\log 3$ and we obtain

$$\begin{aligned} b'\le 2\max \{|x-x_1|,|y-y_1|\}\le \frac{2}{\log 2} \log u. \end{aligned}$$

That is

$$\begin{aligned} \max \{ \log b' + 0.38, 30\}\le \log \left( 4.22 \log u\right) . \end{aligned}$$

Provided that $\log (4.22 \log u)\ge 30$, Laurent’s lower bound for linear forms in two logarithms yields

$$\begin{aligned} 17.9 (\log 3) \log \left( 4.22 \log u\right) ^2 \ge \log u-\log \Delta -\log 3/2. \end{aligned}$$

If we substitute $u=\Delta (\log \Delta )^{60 \log \log \Delta }$ into this inequality we obtain

$$\begin{aligned} 19.67 \log \left( 4.22 \log \Delta +253.2(\log \log \Delta )^2\right) ^2 \ge 60 (\log \log \Delta )^2-0.41. \end{aligned}$$

Since we assume that $\Delta >10^{80}$ we have $253.2(\log \log \Delta )^2> 37.4 \log \Delta$ and also $0.41< 0.016(\log \log \Delta )^2$. Thus we get

$$\begin{aligned} 19.67 \log \left( 41.62\log \Delta \right) ^2\ge 59.98 (\log \log \Delta )^2, \end{aligned}$$

which does not hold if $\Delta >10^{80}$. Thus Proposition 1 holds under the assumption that $\log (4.22 \log u)\ge 30$, i.e., that $\log u>2.54\cdot 10^{12}$.

Let us assume that $\log u=x\log 2 +y \log 3\le 2.54\cdot 10^{12}$, which implies that $x,y<3.67\cdot 10^{12}$ and therefore we also have $|x-x_1|,|y-y_1|<3.67\cdot 10^{12}$. Assume for the moment that $u\ge \Delta (\log \Delta )^{60 \log \log \Delta }$, then Inequality (17) turns into

$$\begin{aligned} \left| (x-x_1)+(y-y_1)\frac{\log 3}{\log 2}\right| \le \frac{3}{2(\log 2) (\log \Delta )^{60 \log \log \Delta }}<2.39\cdot 10^{-709}. \end{aligned}$$

Note that the 25-th convergent $p_{25}/q_{25}$ to $\frac{\log 3}{\log 2}$ satisfies $p_{25},q_{25}>3.67\cdot 10^{12}$ and we get

$$\begin{aligned} \left| (x-x_1)+(y-y_1)\frac{\log 3}{\log 2}\right|<2.39\cdot 10^{-709}<9.5\cdot 10^{-14} <\left| p_{25}+q_{25}\frac{\log 3}{\log 2}\right| . \end{aligned}$$

But this contradicts the best approximation property of continued fractions (e.g., see [13], Theorem 182). Thus Proposition 1 is proved also in the case that $\log u\le 2.54\cdot 10^{12}$. $\square$

In view of the proof of our main theorems, in particular of Theorem 3, the following corollary will be useful. To state the corollary we write $X=x\log 2+y\log 3$ and $X_1=x_1\log 2+y_1\log 3$. With these notations we have:

### Corollary 2

Assume that $(n,n_1,x,x_1,y,y_1)$ is a solution to (3) with $n\ge 400$ and $n>n_1$. Then we have

$$\begin{aligned} 0.17 \alpha ^n< \exp (X)< 0.448 \alpha ^n (n\log \alpha )^{60 \log (n\log \alpha )} \end{aligned}$$

and

$$\begin{aligned} n\log \alpha -4<X<n\log \alpha +60\log (n\log \alpha ). \end{aligned}$$

### Proof

If $n\ge 400$ then $F_n-F_{n_1}\ge F_{n-2}\ge F_{398}>10^{80}$. Thus writing $\Delta =F_n-F_{n_1}$ Diophantine equations (3) turns into (15) and we apply Proposition 1 with

$$\begin{aligned} \Delta =F_n-F_{n_1}<0.448 \alpha ^n \end{aligned}$$

due to (4). This yields

$$\begin{aligned} \exp (X)=u<0.448 \alpha ^n (n\log \alpha )^{60 \log \log \alpha ^n}<0.448 \alpha ^n (n\log \alpha )^{60 \log (n\log \alpha )}. \end{aligned}$$

On the other hand we have

$$\begin{aligned} 0.17\alpha ^n<0.447\alpha ^n-0.448\alpha ^{n-1}<F_n-F_{n-1}<2^x3^y. \end{aligned}$$

which implies the first inequality. The second inequality in the corollary can easily be deduced by taking logarithms. $\square$

In view of Corollary 2 we want to exclude solutions $(n,n_1,x,x_1,y,y_1)\in {\mathbb {N}}^6$ to (3) with small *n*. Thus we prove next:

### Lemma 11

All solutions $(n,n_1,x,x_1,y,y_1)\in {\mathbb {N}}^6$ to (3) with $n\le 400$ satisfy $F_n-2^x3^y=c\in {\mathcal {C}}_s$.

### Proof

We use the same notations as in the proof of Proposition 1. We set $\Delta =F_n-F_{n_1}<F_{400}<1.77\cdot 10^{83}$. We distinguish now between the following three cases

- $$\log u\ge 2.54\cdot 10^{12}$$,
- $$2\Delta <u$$ and $$\log u < 2.54\cdot 10^{12}$$ and
- $$u\le 2\Delta $$

Firstly, let us assume that $\log u\ge 2.54\cdot 10^{12}$ holds, which also implies $u>2\Delta$ as well as

$$\begin{aligned} \log u=x \log 2+y\log 3>\max \{\log (2\Delta ),\exp (30)/4.22\}. \end{aligned}$$

By the same arguments as in the proof of Proposition 1 we get the inequality

$$\begin{aligned} 17.9 \log 3 \log \left( 4.22 \log u\right) ^2 \ge \log u-\log \Delta -\log 3/2. \end{aligned}$$

Since we assume that $\Delta <1.77 \cdot 10^{83}$ this inequality implies $\log u<1254$ which contradicts our assumption that $\log u\ge 2.54\cdot 10^{12}$ holds. Hence, the first case cannot hold.

Therefore we may assume that $\log u=x\log 2+y\log 3<2.54\cdot 10^{12}$, i.e. that $x,y<3.67\cdot 10^{12}$. Assuming that $u>2 \Delta$, that is we are in the second case, we have by the same arguments as in the proof of Proposition 1 the following inequality:

$$\begin{aligned} \left| (x-x_1)\log 2+(y-y_1)\log 3\right| \le 3/2 \frac{\Delta }{u}, \end{aligned}$$

which is exactly inequality (17). Note that the 25-th convergent $p_{25}/q_{25}$ to $\frac{\log 3}{\log 2}$ satisfies $p_{25},q_{25}>3.67\cdot 10^{12}$ and by the best approximation property of continued fractions (e.g. see [13, Theorem 182]) we get

$$\begin{aligned} 6.59\cdot 10^{-14}<\left| p_{25}\log 2+q_{25}\log 3\right| \le \left| (x-x_1)+(y-y_1)\frac{\log 3}{\log 2}\right| < \frac{2.65 \cdot 10^{83}}{u}. \end{aligned}$$

However, this implies $2^x3^y=u< 4.03 \cdot 10^{96}$ and

$$\begin{aligned} |c|=|F_n-2^x3^y|<4.03 \cdot 10^{33}<10^{96}. \end{aligned}$$

But solutions to (3) correspond to two solutions (*n*, *x*, *y*) and $(n_1,x_1,y_1)$ to (2) for a fixed $c\in {\mathbb {Z}}$. Thus by Theorem 4 we have $c=F_n-2^x3^y\in {\mathcal {C}}_s$.

Finally, let us assume that $u\le 2 \Delta < 3.54 \cdot 10^{83}$. But by assumption we also have $F_n\le F_{400}<1.77 \cdot 10^{83}$. Therefore we deduce $|c|=|F_n-2^x3^y|<3.54 \cdot 10^{83}$, which indicates by the same argument as in the previous case that $c=F_n-2^x3^y\in {\mathcal {C}}_s$ holds.

## An absolute upper bound for *n* in the case that $c>0$

The main purpose of this section is to prove the following Proposition:

### Proposition 2

Assume that $c>0$ and that the Diophantine equation (2) has at least three solutions $(n,x,y),(n_1,x_1,y_1),(n_2,x_2,y_2)$, with $n> n_1>n_2>1$. Then we have $n<5.79\cdot 10^{30}$. In particular we have:

$$\begin{aligned} n<5.79\cdot 10^{30}, \quad x<4.02 \cdot 10^{30}, \quad y<2.54\cdot 10^{30}. \end{aligned}$$

In view of Corollary 2 and Lemma 11 we may assume that $n\ge 400$. The proof of the proposition is rather technical and we split it up into several steps:

**Step 1:** *We show that* $n-n_1\ll \log n$ *holds.*

First, we note that in the case that $c>0$ we have $F_n>2^x3^y$ and $F_{n_1}>2^{x_1}3^{y_1}$. We consider (3) and we rewrite it as

$$\begin{aligned} \frac{\alpha ^n}{\sqrt{5}}-2^x3^y=\frac{\alpha ^{n_1}}{\sqrt{5}}-2^{x_1}3^{y_1} -\frac{\beta ^{n_1}}{\sqrt{5}}+\frac{\beta ^{n}}{\sqrt{5}}. \end{aligned}$$

Since $\left| -\beta ^{n_1}+\beta ^n\right| \le -\beta +\beta ^2=1$ we conclude that

$$\begin{aligned} \left| \frac{\alpha ^{n_1}}{\sqrt{5}}-2^{x_1}3^{y_1} -\frac{\beta ^{n_1}}{\sqrt{5}}+\frac{\beta ^{n}}{\sqrt{5}}\right| \le \frac{\alpha ^{n_1}}{\sqrt{5}} \end{aligned}$$

and we get

$$\begin{aligned} \left| \frac{2^x3^y\sqrt{5}}{\alpha ^n}-1 \right| \le \alpha ^{-(n-n_1)}. \end{aligned}$$

We assume for the moment that $n-n_1\ge 2$, then taking logarithms and applying Lemma 1 we obtain

18 $$\begin{aligned} |\Lambda _1|=|\log \sqrt{5}+x\log 2+y\log 3-n\log \alpha |\le \frac{3}{2} \alpha ^{-(n-n_1)}. \end{aligned}$$

Since $\sqrt{5},2,3$ and $\alpha$ are multiplicatively independent, we conclude $\Lambda _1\ne 0$. We apply Matveev’s theorem (Lemma 2) and note that

$$\begin{aligned} h'(2)=2\log 2, \quad h'(3)=2\log 3, \quad h'(\sqrt{5}) =\log 5, \quad h'(\alpha )=\log (\alpha ), \end{aligned}$$

i.e. $\Omega \simeq 2.3591\dots$ and due to (5) we have

$$\begin{aligned} B=\max \left\{ \frac{\log 5}{\log \alpha },x\frac{\log 2}{\log \alpha },y \frac{\log 3}{\log \alpha },n \right\} =n. \end{aligned}$$

Therefore we obtain

$$\begin{aligned} 8.123 \cdot 10^{13} \log (13.81 n)>(n-n_1) \log \alpha -\log (1.5) \end{aligned}$$

which yields the statement of Step 1:

### Lemma 12

Assume that $c>0$, then we have

$$\begin{aligned} n-n_1<1.69 \cdot 10^{14} \log (13.81 n). \end{aligned}$$

**Step 2:** *We show that* $X-X_1 \ll (\log n)^2$ *holds.*

In Step 2 we rewrite (3) into the following form

$$\begin{aligned} \frac{\alpha ^n-\alpha ^{n_1}}{\sqrt{5}}-2^x3^y=-2^{x_1}3^{y_1} +\frac{\beta ^n-\beta ^{n_1}}{\sqrt{5}} \end{aligned}$$

and get

$$\begin{aligned} \left| \frac{\alpha ^{n_1}(\alpha ^{n-n_1}-1)}{2^x3^y\sqrt{5}}-1\right| \le \frac{2^{x_1}3^{y_1}}{2^x3^y}+\frac{\beta ^n-\beta ^{n_1}}{2^x3^y \sqrt{5}}<1.45 \exp (-(X-X_1)). \end{aligned}$$

Assuming that $X-X_1>1.1$ we have $\frac{1}{2}>1.45 \exp (-(X-X_1))$ and taking logarithms we get

19 $$\begin{aligned} |\Lambda _2|&:=\left| n_1\log \alpha +\log \left( \frac{\alpha ^{n-n_1}-1}{\sqrt{5}}\right) -x \log 2 -y\log 3\right| \nonumber \\&<2.2\exp (-(X-X_1)) \end{aligned}$$

Let us note that $\Lambda _2=0$ holds exactly when

$$\begin{aligned} \frac{\alpha ^{n_1}(\alpha ^{n-n_1}-1)}{2^x3^y\sqrt{5}}=1. \end{aligned}$$

But then also the algebraic conjugate of the right hand side of the equation yields

$$\begin{aligned} 1=\left| \frac{\beta ^{n_1}(\beta ^{n-n_1}-1)}{2^x3^y\sqrt{5}}\right| <1 \end{aligned}$$

an immediate contradiction and we deduce that $\Lambda _2\ne 0$.

Now we may apply Matveev’s theorem (Lemma 2). Similar as above we can take $B=n$ and using formula (7) we get by applying the results of Step 1:

$$\begin{aligned} h\left( \frac{\alpha ^{n-n_1}-1}{\sqrt{5}}\right)<\frac{(n-n_1) \log \alpha }{2}+\log 2+h(\sqrt{5})<4.07 \cdot 10^{13} \log (13.81 n). \end{aligned}$$

Therefore Matveev’s theorem (Lemma 2) applied to (19) yields

$$\begin{aligned} 2.573 \cdot 10^{26} \left( \log (13.81 n)\right) ^2>X-X_1-\log 2.2 \end{aligned}$$

and we get:

### Lemma 13

Assume that $c>0$, then we have

$$\begin{aligned} X-X_1<2.58 \cdot 10^{26} \left( \log (13.81 n)\right) ^2. \end{aligned}$$

Let us write $x_{min}=\min \{x,x_1\}$ and $y_{min}=\min \{y,y_1\}$, then we show next:

**Step 3:** *We show that* $x_{min},y_{min} \ll (\log n)^3$.

We rewrite Equation (3) and get

$$\begin{aligned} \frac{\alpha ^{n_1}(\alpha ^{n-n_1}-1)}{\beta ^{n_1}(\beta ^{n-n_1}-1)}-1 =\frac{2^{x_{min}}3^{y_{min}}{\mathop {\overbrace{(2^{x-x_{min}} 3^{y-y_{min}}-2^{x_1-x_{min}}3^{y_1-y_{min}})}}\limits ^{:=A}}\beta ^{-n_1}\sqrt{5}}{\beta ^{n-n_1}-1}. \end{aligned}$$

Since the 2-adic and 3-adic valuations of $\beta$, *A* and $\sqrt{5}$ are zero we obtain

20 $$\begin{aligned} v_2\left( \left( \frac{\alpha }{\beta }\right) ^{n_1} \frac{\alpha ^{n-n_1}-1}{\beta ^{n-n_1}-1}-1\right) =x_{min}-v_2(\beta ^{n-n_1}-1) \end{aligned}$$

and

21 $$\begin{aligned} v_3\left( \left( \frac{\alpha }{\beta }\right) ^{n_1} \frac{\alpha ^{n-n_1}-1}{\beta ^{n-n_1}-1}-1\right) =y_{min}-v_3(\beta ^{n-n_1}-1). \end{aligned}$$

First, we want to estimate $v_p(\beta ^{n-n_1}-1)$ for $p=2,3$. By Lemma 6 we have

$$\begin{aligned} v_p(\beta ^{n-n_1}-1)&\le 1+\frac{\log (n-n_1)}{\log p}\\&<1+\frac{\log (1.69 \cdot 10^{14} \log (13.81 n))}{\log p}\\&<1+\frac{19.51 \log \log n}{\log p}, \end{aligned}$$

under the assumption that $n>400$.

Now let us estimate the left hand side of (20) and (21) respectively. If we assume that $n-n_1$ is even (see Lemma 5) we have

$$\begin{aligned} v_p\left( \left( \frac{\alpha }{\beta }\right) ^{n_1} \frac{\alpha ^{n-n_1}-1}{\beta ^{n-n_1}-1}-1\right) =v_p(\pm \alpha ^{2n_1+n-n_1}-1)< 1+\frac{\log (2n)}{\log p} \end{aligned}$$

and we get the inequalities

$$\begin{aligned} x_{min}< 1+\frac{\log (2n)}{\log 2}+1+\frac{19.51 \log \log n}{\log 2}<10.4 \log n \end{aligned}$$

and

$$\begin{aligned} y_{min}< 1+\frac{\log (2n)}{\log 3}+1+\frac{19.51 \log \log n}{\log 3}<6.7 \log n \end{aligned}$$

The case that $n-n_1=1$ or 3 can be treated similarly and we get even smaller bounds for $x_{min}$ and $y_{min}$.

In the case that $n-n_1$ is odd and $\ne 1,3$ we apply Lemma 4 to the right hand side of (20) and (21) respectively. In this case $\alpha _1=\frac{\alpha }{\beta }$ and $\alpha _2=\frac{\alpha ^{n-n_1}-1}{\beta ^{n-n_1}-1}$ are multiplicatively independent (see Lemma 5) and we have $h(\alpha _1)=2h(\alpha )=\log \alpha$, thus we choose $h'(\alpha _1)=\log \alpha$ in case of (20) and $h'(\alpha _1)=\log \sqrt{3}$ in case of (21). We also have

$$\begin{aligned} h(\alpha _2)\le 2h(\alpha ^{n-n_1}-1)< (n-n_1) \log \alpha +\log 4<8.14\cdot 10^{13} \log (13.81 n). \end{aligned}$$

Therefore we have with $A_1=h'(\alpha _1)$ and $A_2=8.14\cdot 10^{13} \log (13.81 n)$ the estimate

$$\begin{aligned} E'=\frac{b_1}{A_2}+\frac{b_2}{A_1}<\frac{1}{A_2}+\frac{n_1}{A_1}<\frac{n}{A_1}. \end{aligned}$$

Assuming that $n>11000$ we get

$$\begin{aligned} E&=\max \left\{ \log E'+\log \log p+0.4,10,10\log p\right\} \\&<\log n-\log h'(\alpha _1)+\log \log p+0.4\\&<\log n \end{aligned}$$

in any case. Therefore we get

$$\begin{aligned} x_{min}&<\frac{24 pg}{(p-1)(\log p)^4}E^2D^4h' (\alpha _1)h'(\alpha _2)+v_2(\beta ^{n-n_1}-1)\\&<5.63\cdot 10^{17} (\log n)^3 \end{aligned}$$

under the assumption that $n>11000$ and since $p=2$ implies $g\le 3$. Similarly we obtain

$$\begin{aligned} y_{min}<2.04\cdot 10^{17} (\log n)^3. \end{aligned}$$

Altogether we obtain the following lemma:

### Lemma 14

Assume that $c>0$, then we have that either

$$\begin{aligned} x_{min}<5.63\cdot 10^{17} (\log n)^3\quad \text {and} \quad y_{min}<2.04\cdot 10^{17} (\log n)^3. \end{aligned}$$

or $$n<11000$$ holds.

**Step 4:** *Under the assumption that a third solution* $(n_2,x_2,y_2)$ *with* $n>n_1>n_2$ *exists, we deduce that* $n\ll (\log n)^3$, *which yields an absolute bound for* *n*.

Assume that $n>11000$. We have to consider three subcases to deal with Step 4:

*Case I:* We assume that $x_{min}=x_1$ and $y_{min}=y_1$.

In this case we have due to Lemma 14

$$\begin{aligned} X_1=x_1 \log 2+y_1\log 3=x_{min} \log 2+y_{min}\log 3< 6.15\cdot 10^{17} (\log n)^3. \end{aligned}$$

In view of Lemma 13 and Corollary 2 we get

22 $$\begin{aligned} n\log \alpha -4&<X=X_1+(X-X_1)\nonumber \\&<6.15\cdot 10^{17} (\log n)^3+2.58 \cdot 10^{26} \left( \log (13.81 n)\right) ^2. \end{aligned}$$

Solving inequality (22) we get $n<2.84\cdot 10^{30}$. Substituting this bound for *n* in (22) we get an upper bound for *X* and therefore also for *x* and *y*, namely $x<1.97\cdot 10^{30}$ and $y<1.25\cdot 10^{30}$

*Case II:* We assume that $x_{min}=x_1$ and $y_{min}=y$.

To resolve this case we have to assume that a third solution $(n_2,x_2,y_2)$ to (2) exists. Let us write $x'_{min}=\min \{x,x_2\}$ and $y'_{min}=\min \{y,y_2\}$. Assuming that $x'_{min}=x_2$ and $y'_{min}=y_2$, we end up in the same situation as in Case I and obtain the same upper bounds for *n*, *x* and *y*.

Let us note that by an application of Lemma 14 to each of the three solutions $(n,n_1,x,x_1,y,y_1)$, $(n,n_2,x,x_2,y,y_2)$ and $(n_1,n_2,x_1,x_2,y_1,y_2)$ to (3), we have that two quantities out of $x,x_1,x_2$ are smaller than $5.63\cdot 10^{17} (\log n)^3$ and two quantities out of $y,y_1,y_2$ are smaller than $2.04 \cdot 10^{17} (\log n)^3$.

Assume for the moment that $x_2>x$. Then we have

$$\begin{aligned} x<5.63\cdot 10^{17} (\log n)^3 \end{aligned}$$

and since $y=y_{min}$ we also have

$$\begin{aligned} y<2.04 \cdot 10^{17} (\log n)^3. \end{aligned}$$

Thus we get

$$\begin{aligned} n\log \alpha -4<X<6.15\cdot 10^{17} (\log n)^3 \end{aligned}$$

which yields an even smaller bound for *n* as we obtained in Case I.

Therefore we may assume that $x>x_1,x_2$, which yields

$$\begin{aligned} x_1,x_2<5.63\cdot 10^{17} (\log n)^3. \end{aligned}$$

But either $y_1$ or $y_2$ is bounded by $2.04 \cdot 10^{17} (\log n)^3$, which yields in any case

$$\begin{aligned} X_2=x_2\log 2+y_2\log 3<6.15\cdot 10^{17} (\log n)^3. \end{aligned}$$

Note that $X_2<X_1$ holds, since by assumption we have $n_2<n_1$ and therefore we have

$$\begin{aligned} 0<F_{n_1}-F_{n_2}=\exp (X_1)-\exp (X_2). \end{aligned}$$

Therefore we get

$$\begin{aligned} n\log \alpha -4&<X=X_2+(X_1-X_2)+(X-X_1)\\&<6.15\cdot 10^{17} (\log n)^3+5.16 \cdot 10^{26} \left( \log (13.81 n)\right) ^2. \end{aligned}$$

which yields, by a similar argument as in Case I, the upper bounds

$$\begin{aligned} n<5.79\cdot 10^{30}, \quad x< 4.02 \cdot 10^{30}, \quad y< 2.54\cdot 10^{30}. \end{aligned}$$

*Case III:* We assume that $x_{min}=x$ and $y_{min}=y_1$.

By exchanging the roles of *x* and *y* we can resolve this case by the same arguments as used in the resolution of Case II.

### Remark 2

Let $c_x>x_{min}$, $c_y>y_{min}$ and $c_X>X-X_1$ be upper bounds respectively. Then we showed in Step 4 that

23 $$\begin{aligned} X<c_x \log 2 +c_y \log 3 +2 c_X \end{aligned}$$

holds.

## An absolute upper bound for *n* in the case that $c<0$

The main purpose of this section is to prove the following Proposition:

### Proposition 3

Assume that $c<0$ and that the Diophantine equation (2) has at least four solutions $(n,x,y),(n_1,x_1,y_1),(n_2,x_2,y_2),(n_3,x_3,y_3)$, with $n> n_1>n_2>n_3>1$. Then we have $n<6.71\cdot 10^{24}$. In particular we have:

$$\begin{aligned} n<6.71\cdot 10^{24}, \quad x< 4.66\cdot 10^{24}, \quad y< 2.94 \cdot 10^{24}. \end{aligned}$$

In view of Corollary 2 and Lemma 11 we may assume that $n\ge 400$. The proof of the proposition is rather technical and again we split it up into several steps:

**Step 1:** *We show that* $X-X_1 \ll (\log n)$ *holds.*

First we note that since $c<0$ we have $F_n-2^x3^y\le -1$ and therefore we have $0<2^x3^y-\frac{\alpha ^n}{\sqrt{5}}$. Similarly as in Step 1 in the proof of Proposition 2 we rewrite (3) and get

$$\begin{aligned} 0<2^x3^y-\frac{\alpha ^n}{\sqrt{5}}=2^{x_1}3^{y_1} -\frac{\alpha ^{n_1}}{\sqrt{5}}+\frac{\beta ^{n_1}}{\sqrt{5}} -\frac{\beta ^{n}}{\sqrt{5}}<2^{x_1}3^{y_1}. \end{aligned}$$

Dividing through $2^x3^y$ we get

$$\begin{aligned} \left| \frac{\alpha ^n}{2^x3^y \sqrt{5}} -1\right| <\exp (X_1-X). \end{aligned}$$

Assuming that $X-X_1>1$ and taking logarithms we get

24 $$\begin{aligned} |\Lambda _1|=|n\log \alpha -x \log 2 -y\log 3 -\log \sqrt{5}|<1.5\exp (-(X-X_1)). \end{aligned}$$

As in Step 1 in the proof of Proposition 2 we have $\Lambda _1\ne 0$ since $\alpha ,2,3$ and $\sqrt{5}$ are multiplicatively independent and we also can choose $B=n$. Thus we get by an application of Matveev’s theorem (Lemma 2) to (24):

$$\begin{aligned} 8.123 \cdot 10^{13} \log (13.81 n)>(X-X_1)-\log (1.5). \end{aligned}$$

Hence we get the following lemma:

### Lemma 15

Assume that $c<0$, then we have

$$\begin{aligned} X-X_1<8.13 \cdot 10^{13} \log (13.81 n). \end{aligned}$$

**Step 2:** *We show that* $n-n_1 \ll (\log n)^2$ *holds, provided that a third solution* $(n_2,x_2,y_2)$ *with* $n>n_1>n_2$ *exists.*

Since we assume that a third solution exists, Corollary 2 also holds for $n_1$ and moreover due to Lemma 11 we may also assume that $n_1\ge 400$. Therefore we have by Corollary 2

$$\begin{aligned} 0.17 \alpha ^{n_1}< \exp (X_1)=2^{x_1}3^{y_1}< 0.448 \alpha ^{n_1} (n_1\log \alpha )^{60 \log (n_1\log \alpha )}. \end{aligned}$$

With this inequality in mind we obtain now

$$\begin{aligned} 2^x3^y-\frac{\alpha ^n}{\sqrt{5}}&=2^{x_1}3^{y_1}-\frac{\alpha ^{n_1}}{\sqrt{5}} +\frac{\beta ^{n_1}}{\sqrt{5}}-\frac{\beta ^{n}}{\sqrt{5}}\\&<2^{x_1}3^{y_1}<0.448 \alpha ^{n_1} (n_1\log \alpha )^{60 \log (n_1\log \alpha )}. \end{aligned}$$

Dividing throug $\alpha ^n/\sqrt{5}$ we obtain

$$\begin{aligned} \left| \frac{\alpha ^n}{2^x3^y \sqrt{5}} -1\right| <1.01 \alpha ^{n_1-n} (n_1\log \alpha )^{60 \log (n_1\log \alpha )}. \end{aligned}$$

In view of an application of Lemma 1 we have to assume that

$$\begin{aligned} 1.01 \alpha ^{n_1-n} (n_1\log \alpha )^{60 \log (n_1\log \alpha )}<0.5. \end{aligned}$$

This inequality is certainly satisfied if

$$\begin{aligned} n-n_1>\frac{60\log (n \log \alpha )^2+\log 2.02}{\log \alpha }:=\tilde{c}(n). \end{aligned}$$

Assuming $n-n_1>\tilde{c}(n)$ and taking logarithms we obtain

$$\begin{aligned} |\Lambda _1|=|n\log \alpha -x \log 2 -y\log 3-\log \sqrt{5}|<1.52 \alpha ^{n_1-n} (n_1\log \alpha )^{60 \log (n_1\log \alpha )} \end{aligned}$$

and an application of Matveev’s theorem yields

$$\begin{aligned} 8.123 \cdot 10^{13} \log (13.81 n)>(n-n_1)\log \alpha -60\log (n \log \alpha )^2-\log 3.03. \end{aligned}$$

Since we assume that $n>400$ we obtain

$$\begin{aligned} n-n_1< 3.48\cdot 10^{13} \log (n\log \alpha )^2. \end{aligned}$$

Since this bound is larger than $\tilde{c}(n)$ we get:

### Lemma 16

Assume that $c<0$ and that (2) has at least three solutions. Then we have

$$\begin{aligned} n-n_1<3.48\cdot 10^{13} \log (n\log \alpha )^2. \end{aligned}$$

**Step 3:** *We show that under the assumption that a third solution* $(n_2,x_2,y_2)$ *with* $n>n_1>n_2$ *to* (2) *exists, we have* $x_{min},y_{min} \ll (\log n)^4$.

We follow the same arguments as in Step 3 in the proof of Proposition 2 but using the bounds from Lemma 16 instead of Lemma 13. In particular, we consider again the *p*-adic valuations (20) and (21).

Let us estimate $v_p(\beta ^{n-n_1}-1)$ for $p=2,3$. In our situation we obtain by Lemma 6 and our bounds for $n-n_1$ the upper bound

$$\begin{aligned} v_p(\beta ^{n-n_1}-1)&\le 1+\frac{\log (n-n_1)}{\log p}\\&<1+\frac{\log (3.48\cdot 10^{13} \log (n\log \alpha )^2)}{\log p}\\&<1+\frac{19.46 \log \log n}{\log p}, \end{aligned}$$

under the assumption that $n>400$.

Assuming that $n-n_1$ is even or $=1,3$ we apply Lemma 6 and get by the same argument as in the $c>0$ case that

$$\begin{aligned} v_p\left( \left( \frac{\alpha }{\beta }\right) ^{n_1} \frac{\alpha ^{n-n_1}-1}{\beta ^{n-n_1}-1}-1\right) =v_p (\pm \alpha ^{2n_1+n-n_1}-1)< 1+\frac{\log (2n)}{\log p}. \end{aligned}$$

Hence we get the inequalities

$$\begin{aligned} x_{min}< 1+\frac{\log (2n)}{\log 2}+1+\frac{19.46 \log \log n}{\log 2}<10.4 \log n \end{aligned}$$

and

$$\begin{aligned} y_{min}< 1+\frac{\log (2n)}{\log 3}+1+\frac{19.46 \log \log n}{\log 3}<6.7 \log n. \end{aligned}$$

In the case that $n-n_1$ is odd and $\ne 1,3$ we apply Lemma 4. We note that the only difference between the case $c>0$ and $c<0$ is that we have different upper bounds for $n-n_1$ which results in different upper bounds for $h(\alpha _2)$. In particular, we have in this case

$$\begin{aligned} h(\alpha _2)\le 2h(\alpha ^{n-n_1}-1)< (n-n_1)\log \alpha +\log 4<1.68\cdot 10^{13} \log (n\log \alpha )^2. \end{aligned}$$

Hence, we have as before with $A_1=h'(\alpha _1)$ and $A_2=1.68\cdot 10^{13} \log (n\log \alpha )^2$

$$\begin{aligned} E'=\frac{b_1}{A_2}+\frac{b_2}{A_1}<\frac{1}{A_2}+\frac{n_1}{A_1}<\frac{n}{A_1} \end{aligned}$$

and assuming that $n>11000$ we also get $E<\log n$. Therefore we get

$$\begin{aligned} x_{min}< 9.42\cdot 10^{16} (\log n)^4 \end{aligned}$$

and

$$\begin{aligned} y_{min}< 2.15\cdot 10^{17} (\log n)^4. \end{aligned}$$

Thus we obtain the following lemma:

### Lemma 17

Assume that $c<0$, $n>11000$ and that (2) has at least three solutions, then we have

$$\begin{aligned} x_{min}=\min \{x,x_1\}<9.42\cdot 10^{16} (\log n)^4 \end{aligned}$$

and

$$\begin{aligned} y_{min}=\min \{y,y_1\}<2.15\cdot 10^{17} (\log n)^4. \end{aligned}$$

**Step 4:** *Under the assumption that a third solution* $(n_2,x_2,y_2)$ *and forth solution* $(n_3,x_3,y_3)$ *with* $n>n_1>n_2>n_3$ *exists, we deduce that* $n\ll (\log n)^4$, *which yields an absolute bound for* *n*.

In order to deduce upper bounds for $x'_{min}=\min \{x,x_2\}$ and $y'_{min}=\min \{y,y_2\}$ and to find upper bounds for the two smaller ones out of $x,x_1,x_2$ and $y,y_1,y_2$ respectively, we have to assume the existence of a fourth solution.

Thus the same arguments as in the case that $c>0$ holds can be applied. Therefore we get due to Remark 2 together with Corollary 2

$$\begin{aligned} n\log \alpha -4<X&<x_{min}\log 2+y_{min}\log 3+2c_X\\&<3.02 \cdot 10^{17} (\log n)^4+1.63 \cdot 10^{14} \log (13.81 n), \end{aligned}$$

where $c_X$ is the upper bound for $X-X_1$ and $X_1-X_2$ implied by Lemma 15. Solving this inequality for *n* we find that $n<6.71\cdot 10^{24}$. The same inequality yields then $X<3.23\cdot 10^{24}$ and we also find absolute upper bounds for *x* and *y* which concludes the proof of Proposition 3.

## Reduction of the upper bound, if $c>0$

We follow the steps in the proof of Proposition 2 and instead of applying lower bounds for linear forms in logarithms we use LLL-reduction or the *p*-adic reduction method due to Pethő and de Weger [17].

**Step 1:** *We show that* $n-n_1\le 311$ *holds.*

Assuming that $n-n_1\ge 2$ we obtain the inequality (18), which is

$$\begin{aligned} |\Lambda _1|=|\log \sqrt{5}+x\log 2+y\log 3-n\log \alpha |<3/2 \alpha ^{-(n-n_1)}. \end{aligned}$$

We consider the approximation lattice

$$\begin{aligned} {\mathcal {A}}=\begin{pmatrix} 1 &{} 0 &{} 0 \\ 0 &{} 1 &{} 0 \\ \lfloor { C \log 2}\rfloor &{} \lfloor { C\log 3}\rfloor &{} \lfloor { C\log \alpha }\rfloor \end{pmatrix} \end{aligned}$$

with $C=10^{96}$ and choose $y=(0,0,-\lfloor C \log \sqrt{5}\rfloor )$. An application of Lemma 7 yields

$$\begin{aligned} l({\mathcal {L}},y):=c_1=1.7059603247874 \dots \cdot 10^{17}. \end{aligned}$$

Moreover, we have due to Proposition 2

$$\begin{aligned} x<X_1=4.02 \cdot 10^{30}, \quad y<X_2=2.54 \cdot 10^{30}, \quad n<X_3=5.79 \cdot 10^{30}. \end{aligned}$$

With this choice of parameters we can apply Lemma 8, since $c_1^2\ge T^2+S$ is satisfied and by choosing $c_3=1.5$ and $c_4=\log \alpha$ we get $n-n_1 \le 311$.

**Step 2:** *We show that* $X-X_1<178.46$ *holds.*

In this step we consider inequality (19), which is

$$\begin{aligned} |n_1\log \alpha +\log \left( \frac{\alpha ^{n-n_1}-1}{\sqrt{5}}\right) -x \log 2 -y\log 3|<2.2\exp (-(X-X_1)) \end{aligned}$$

and use the same approximation lattice as in Step 1

$$\begin{aligned} {\mathcal {A}}=\begin{pmatrix} 1 &{} 0 &{} 0 \\ 0 &{} 1 &{} 0 \\ \lfloor { C \log 2}\rfloor &{} \lfloor { C\log 3}\rfloor &{} \lfloor { C\log \alpha }\rfloor \end{pmatrix} \end{aligned}$$

but with $C=10^{110}$. Let us write $t=n-n_1$, then for each $t=1$ to 311 we choose $y=(0,0,-\lfloor C \log \frac{\alpha ^t-1}{\sqrt{5}}\rfloor )$. Let us note that for all 311 values of *t* the constant *C* is large enough such that the LLL-reduction process works and we finally obtain $X-X_1<178.13$ in each case.

**Step 3:** *We show that* $x_{min}\le 113$ *and* $y_{min}\le 75$ *holds.*

We consider (20) and (21) that is

$$\begin{aligned} z_2:=v_2\left( \left( \frac{\alpha }{\beta }\right) ^{n_1} \frac{\alpha ^{n-n_1}-1}{\beta ^{n-n_1}-1}\right) =x_{min}-v_2(\beta ^{n-n_1}-1) \end{aligned}$$

and

$$\begin{aligned} z_3:=v_3\left( \left( \frac{\alpha }{\beta }\right) ^{n_1} \frac{\alpha ^{n-n_1}-1}{\beta ^{n-n_1}-1}\right) =y_{min}-v_3(\beta ^{n-n_1}-1). \end{aligned}$$

First, we note that by Lemma 6 we have

$$\begin{aligned} v_p(\beta ^{n-n_1}-1)\le 1+\frac{\log (n-n_1)}{\log p} <\left\{ \begin{array}{cc} 9.3 &{} \quad \text {if}\;\;p=2;\\ 6.3 &{} \quad \text {if}\;\;p=3. \end{array}\right. \end{aligned}$$

Let us consider the case that $n-n_1$ is even, then we have

$$\begin{aligned} \left( \frac{\alpha }{\beta }\right) ^{n_1} \frac{\alpha ^{n-n_1}-1}{\beta ^{n-n_1}-1}-1=\pm \alpha ^{2n_1+n-n_1}-1 \end{aligned}$$

and therefore we get by Lemma 6

$$\begin{aligned} v_p\left( \left( \frac{\alpha }{\beta }\right) ^{n_1} \frac{\alpha ^{n-n_1}-1}{\beta ^{n-n_1}-1}-1\right)< 1+\frac{\log (2n)}{\log p}<\left\{ \begin{array}{cc} 104.2 &{} \quad \text {if}\;\;p=2;\\ 66.2 &{} \quad \text {if}\;\;p=3. \end{array}\right. \end{aligned}$$

which yields

$$\begin{aligned} x_{min}\le 113 \quad \text {and}\quad y_{min}\le 72. \end{aligned}$$

Let us note that in the case that $n-n_1=1$ or 3 we get even smaller bounds for $x_{min}$ and $y_{min}$.

Let us consider now the case that $n-n_1$ is odd and $\ne 1,3$. Let us write

$$\begin{aligned} \tau (t)=\frac{\alpha ^t-1}{\beta ^t-1}. \end{aligned}$$

Let us assume that $z_2>2$ and $z_3>1$. That is we have

$$\begin{aligned} \left| \left( \frac{\alpha }{\beta }\right) ^{n_1}\tau (t)-1 \right| _2=2^{-z_2}<\frac{1}{2} \end{aligned}$$

and

$$\begin{aligned} \left| \left( \frac{\alpha }{\beta }\right) ^{n_1}\tau (t)-1 \right| _3=3^{-z_3}<\frac{1}{\sqrt{3}} \end{aligned}$$

and therefore we may apply (9). Thus we have due to the properties of *p*-adic logarithms

25 $$\begin{aligned} z_p=v_p\left( \left( \frac{\alpha }{\beta }\right) ^{n_1}\tau (t)-1\right) =v_p(n_1\log _p(\alpha /\beta )+\log _p(\tau (t))), \end{aligned}$$

with $p=2,3$. We distinguish between two cases:

*Case I:* In this case we assume that $\log _p (\tau (t)) = 0$, which seems to be rather unlikely, unless $t=0$. However, in this case equation (25) turns into

$$\begin{aligned} z_p&=v_p ( n_1 \log _p (\alpha /\beta ) )\\&\le v_p ( n) + v_p( \log _p (\alpha /\beta ) )\\&= \left\{ \begin{array}{lc} v_2(n)+2<104.2 &{} \quad \text {if}\;\;p=2;\\ v_3(n)+1<65.5 &{} \quad \text {if}\;\;p=3. \end{array}\right. \end{aligned}$$

which yields

$$\begin{aligned} x_{min}\le 113 \quad \text {and}\quad y_{min}\le 71. \end{aligned}$$

*Case II:* Now, we consider the case that $\log _p (\tau (t)) \ne 0$. Since $\alpha$ and $\beta$ are conjugate also $\log _p \alpha$ and $\log _p \beta$ are conjugate, and we deduce that $\log _p(\alpha /\beta ) = \log _p \alpha - \log _p \beta \in \sqrt{5}{\mathbb {Q}}_p$. By a similar argument we get $\log _p (\tau (t)) \in \sqrt{5}{\mathbb {Q}}_p$. Thus, we have

$$\begin{aligned} \zeta = \frac{\log _p (\tau (t))}{\log _p(\alpha /\beta )} = \sum _{n=k}^\infty u_np^n \in {\mathbb {Q}}_p. \end{aligned}$$

for some $k\in {\mathbb {Z}}$. In the case that $k<0$ we have due to (25)

$$\begin{aligned} z_p&= v_p(n_1\log _p(\alpha /\beta )+\log _p(\tau (t)))\\&=v_p\left( \log _p(\alpha /\beta ) \cdot (n_1+\log _p(\tau (t))/\log _p(\alpha /\beta ))\right) \\&=v_p(\log _p((\alpha /\beta )))+{\mathop {\overbrace{v_p(n_1+\zeta )}}\limits ^{=0}}. \end{aligned}$$

Note that by assumption $v_p(\zeta )<0$ and $v_p(n_1)\ge 0$, hence that indeed $v_p(n_1+\zeta )=0$. All together this yields an even smaller bounds for $z_p$ as we get in Case I. Note that this is the case for $p=2$ and all odd $1\le t\le 311$. Thus we get in Case II $x_{min}=2$ and therefore $x_{min}\le 113$ in total.

Therefore we may assume that $p=3$ and that $k\ge 0$, i.e. we have

$$\begin{aligned} \zeta =u_0+u_1p+u_2p^2+\cdots \in {\mathbb {Z}}_p \end{aligned}$$

Let $N=5.79\cdot 10^{30}$ our upper bound for *n*. Moreover, let *r* be the smallest possible exponent such that $p^r > N$ and let $0 \le m_0 < p^r$ be the unique integer such that $m_0 \equiv \zeta \mod p^r$. Moreover, let *R* be the smallest index $\ge r$ such that $u_R \ne 0$, if such an index exists. Then we get

$$\begin{aligned} z_p&= v_p ( \log _p (\tau (t))) + n_1 \log _p (\alpha /\beta )) \\&= v_p(\log _p (\alpha /\beta ) )+v_p ( \zeta + n_1)\\&\le v_p(\log _p (\alpha /\beta ) )+ v_p( \zeta -m_0)\\&= v_p(\log _p (\alpha /\beta ) )+v_p(u_Rp^R+\cdots )\\&= v_p(\log _p (\alpha /\beta ) )+R. \end{aligned}$$

That is we choose $r=65$ and for all odd $1\le t\le 311$ with $t\ne 3$ we obtain $R\le 68$. Therefore we have $z_3\le 69$ which yields

$$\begin{aligned} x_{min}\le 113 \quad \text {and}\quad y_{min}\le 75. \end{aligned}$$

**Step 4:** *We find a smaller upper bound for* *n*.

We know by Remark 2 that by writing $c_X$ for an upper bound for $X-X_1$ we have

$$\begin{aligned} X<x_{min}\log 2+y_{min}\log 3+2c_X, \end{aligned}$$

which yields in view of our previous reduction steps the bound $X<516.99$, which implies $x\le 745$ and $y\le 470$. On the other hand we have due to Corollary 2 the inequality

$$\begin{aligned} n\log \alpha -4<X<516.99, \end{aligned}$$

which yields $$n\le 1082$$.

Unfortunately this bound for *n* is too large to obtain a contradiction in view of Lemma 11. However, we can apply the whole reduction process once again, but with the much smaller upper bounds

$$\begin{aligned} n\le 1084,\quad x\le 746,\quad y\le 471. \end{aligned}$$

We obtain:

- $$n-n_1\le 46$$ in Step 1;
- $$X < 34.06$$ in Step 2;
- $$x_{min}\le 18$$ and $$y_{min}\le 16$$,
- which results in $X<98.2$ and therefore $n\le 212$.

However the case that $n\le 212$ has been excluded by Lemma 11 and therefore the proof of Theorem 2 is complete.

## Reduction of the upper bound, if $c<0$

Similar as in Sect. 9 we follow the steps in the proof of Proposition 3. However, before we can start we use continued fractions to have a sharper upper bound for $2^x3^y$ than the bound provided by Proposition 1. Therefore we start with:

**Step Preparation:** *We show that* $2^x3^y\le 1.72\cdot 10^{26}\alpha ^n$ *holds.*

Under the assumption that $n>n_1$, we consider the equation respectively inequality

$$\begin{aligned} 0<2^x3^y-2^{x_1}3^{y_1}=F_n-F_{n_1}<0.448 \alpha ^n. \end{aligned}$$

We divide through $2^x3^y$ and take logarithms. By assuming that $2^x3^y>\alpha ^n$ and by an application of Lemma 1 we get the inequality

$$\begin{aligned} \left| (x-x_1)\log 2+(y-y_1)\log 3\right| < 0.68 \frac{\alpha ^n}{2^x3^y}. \end{aligned}$$

Since we assume that $x<4.66\cdot 10^{24}$ and $y< 2.94 \cdot 10^{24}$ we can use the best approximation property of continued fractions to find a lower bound for the left hand side of the inequality. In particular taking the 48-th convergent to $\mu =\frac{\log 2}{\log 3}$ and apply the best approximation property of continued fractions (see [13, Theorem 182]) we get

$$\begin{aligned} 3.97\cdot 10^{-27}<\left| (x-x_1)\log 2+(y-y_1)\log 3\right| < 0.68 \frac{\alpha ^n}{2^x3^y}, \end{aligned}$$

which yields $$2^x3^y\le 1.72\cdot 10^{26} \alpha ^n$$.

**Step 1:** *We show that* $X-X_1<125.31$ *holds.*

We consider (24) which is

$$\begin{aligned} |n\log \alpha -x \log 2 -y\log 3-\log \sqrt{5}|<1.5\exp (-(X-X_1)). \end{aligned}$$

Therefore we apply as in Step 1 in Sect. 9 the LLL-reduction method on the same lattice but with $C=10^{80}$, $c_3=1.5$ and $c_4=1$ and get $X-X_1<125.31$.

**Step 2:** *We show that under the assumption that a third solution to* (2) *exists* $n-n_1\le 387$ *holds.*

By assuming $c<0$ and by assuming that a third solution exists we obtain

$$\begin{aligned} 0<2^x3^y-\frac{\alpha ^n}{\sqrt{5}}<2^{x_1}3^{y_1}<1.72\cdot 10^{26} \alpha ^{n_1}, \end{aligned}$$

which yields

$$\begin{aligned} \left| \frac{\alpha ^n}{2^x3^y \sqrt{5}} -1\right| < 3.85 \cdot 10^{26}\alpha ^{n_1-n} (n_1\log \alpha )^{60 \log (n_1\log \alpha )}. \end{aligned}$$

In view of an application of Lemma 1 we have to assume that

$$\begin{aligned} 3.85 \cdot 10^{26}\alpha ^{n_1-n}<0.5 \end{aligned}$$

which is certainly satisfied if $n-n_1\ge 128$. Taking logarithms yields

$$\begin{aligned} |n\log \alpha -x \log 2 -y\log 3 -\log \sqrt{5}|< 5.78\cdot 10^{26} \alpha ^{n_1-n}. \end{aligned}$$

As in Step 1 we apply now the LLL-reduction method to the same approximation lattice but in this case we have $c_3=5.78\cdot 10^{26}$ and $c_4=\log \alpha$ and we get $n-n_1\le 387$.

**Step 3:** *We show that under the assumption that a third solution* $(n_2,x_2,y_2)$ *with* $n>n_1>n_2$ *to* (2) *exists, we have* $x_{min}\le 93$ *and* $y_{min}\le 63$.

We follow the exact same steps as in Step 3 of Sect. 9, but with different upper bounds for *n* and $n-n_1$.

First we get

$$\begin{aligned} v_p(\beta ^{n-n_1}-1)\le \left\{ \begin{array}{cc} 9 &{} \quad \text {if}\;\;p=2;\\ 6 &{} \quad \text {if}\;\;p=3. \end{array}\right. \end{aligned}$$

In the case that $n-n_1$ is even or that $n-n_1=3$ we get

$$\begin{aligned} z_p\le \left\{ \begin{array}{cc} 84 &{} \quad \text {if}\;\;p=2;\\ 53 &{} \quad \text {if}\;\;p=3. \end{array}\right. \end{aligned}$$

which yields $x_{min}\le 93$ and $y_{min}\le 59$.

If we consider the case that $n-n_1$ is odd we obtain in Case I again smaller bounds then we found in the $n-n_1$ is even case. However in Case II we have for $p=2$ again $k<0$ in all cases which yields $x_{min}\le 93$. In the case that $p=3$ we use again the method due to Pethő and de Weger [17] and find $z_3\le 57$, which yields $y_{min}\le 63$.

**Step 4:** *We show that* $n\le 806$ *holds.*

Again we use Remark 2 and get

$$\begin{aligned} X<x_{min}\log 2+y_{min}\log 3+2c_X<384.3, \end{aligned}$$

which implies $x\le 554$ and $y\le 349$. Moreover, we have by Corollary 2 the inequality

$$\begin{aligned} n\log \alpha -4<X<384.3, \end{aligned}$$

which yields $$n\le 806$$.

Again this bound for *n* is too large to obtain a contradiction in view of Lemma 11. Thus we apply the whole reduction process once again, but with the much smaller upper bounds

$$\begin{aligned} n\le 806,\quad x\le 554,\quad y\le 349 \end{aligned}$$

instead.

Then we obtain:

- $2^x3^y< 37373.8\alpha ^n$ in the preparation step;
- $$X-X_1< 21.43$$ in Step 1;
- $$n-n_1 \le 65$$ in Step 2;
- $$x_{min}\le 18$$ and $$y_{min}\le 15$$,
- which results in $X< 71.82$ and therefore $n\le 157$.

The bound for *n* is now small enough to apply Lemma 11 which concludes the proof of Theorem 3.

## Appendix

In this appendix we provide the complete list of all solutions to Diophantine equation (2) with $c\in {\mathcal {C}}_s$.

**Table 3:** List of solutions $(n_1,x_1,y_1),\dots ,(n_5,x_5,y_5)$ to (2) with $c\in {\mathcal {C}}_s$ and $n_1\le \dots \le n_5$

*c*	$(n_1,x_1,y_1)$	$(n_2,x_2,y_2)$	$(n_3,x_3,y_3)$	$(n_4,x_4,y_4)$	$(n_5,x_5,y_5)$
$-209719$	(13, 5, 8)	(35, 20, 2)			
$-7768$	(6, 5, 5)	(30, 7, 8)			
$-4606$	(3, 9, 2)	(21, 6, 5)			
$-4574$	(9, 9, 2)	(15, 6, 4)			
$-2291$	(7, 8, 2)	(17, 4, 5)			
$-2215$	(11, 8, 2)	(14, 5, 4)			
$-2043$	(5, 11, 0)	(12, 0, 7)			
$-1694$	(9, 6, 3)	(15, 8, 2)			
$-1118$	(9, 7, 2)	(15, 6, 3)			
$-1063$	(11, 7, 2)	(13, 4, 4)			
$-1011$	(7, 10, 0)	(20, 5, 5)			
$-1003$	(8, 10, 0)	(19, 6, 4)			
$-969$	(4, 2, 5)	(10, 10, 0)			
$-919$	(13, 7, 2)	(14, 4, 4)			
$-775$	(11, 5, 3)	(14, 7, 2)			
$-721$	(6, 0, 6)	(22, 11, 2)			
$-647$	(2, 3, 4)	(14, 10, 0)			
$-640$	(6, 3, 4)	(11, 0, 6)			
$-542$	(9, 6, 2)	(15, 7, 2)			
$-504$	(6, 9, 0)	(12, 3, 4)			
$-487$	(11, 6, 2)	(14, 5, 3)			
$-478$	(6, 1, 5)	(9, 9, 0)			
$-431$	(2, 4, 3)	(10, 1, 5)			
$-427$	(5, 4, 3)	(19, 9, 2)			
$-343$	(11, 4, 3)	(13, 6, 2)			
$-288$	(12, 4, 3)	(24, 6, 6)			
$-254$	(3, 8, 0)	(9, 5, 2)	(15, 5, 3)		
$-253$	(4, 8, 0)	(13, 1, 5)			
$-240$	(4, 0, 5)	(12, 7, 1)			
$-235$	(6, 0, 5)	(8, 8, 0)	(11, 2, 4)		
$-222$	(8, 0, 5)	(9, 8, 0)			
$-199$	(11, 5, 2)	(13, 4, 3)	(14, 6, 2)		
$-190$	(3, 6, 1)	(27, 16, 1)			
$-161$	(2, 1, 4)	(10, 3, 3)			
$-158$	(9, 6, 1)	(15, 8, 1)			
$-154$	(6, 1, 4)	(11, 0, 5)			
$-141$	(4, 4, 2)	(8, 1, 4)			
$-131$	(7, 4, 2)	(17, 6, 3)			
$-127$	(2, 7, 0)	(11, 3, 3)			
$-123$	(5, 7, 0)	(8, 4, 2)			
$-107$	(2, 2, 3)	(8, 7, 0)	(10, 1, 4)		
$-103$	(5, 2, 3)	(11, 6, 1)			
$-95$	(2, 5, 1)	(7, 2, 3)			
$-94$	(3, 5, 1)	(9, 7, 0)			
$-91$	(5, 5, 1)	(13, 2, 4)			
$-73$	(6, 0, 4)	(10, 7, 0)	(11, 1, 4)		
$-62$	(3, 6, 0)	(9, 5, 1)			
$-59$	(5, 6, 0)	(7, 3, 2)			
$-55$	(11, 4, 2)	(13, 5, 2)	(14, 4, 3)		
$-53$	(2, 1, 3)	(10, 2, 3)			
$-51$	(4, 1, 3)	(7, 6, 0)	(8, 3, 2)		
$-47$	(2, 4, 1)	(9, 0, 4)			
$-46$	(3, 4, 1)	(6, 1, 3)			
$-43$	(5, 4, 1)	(8, 6, 0)			
$-41$	(7, 1, 3)	(10, 5, 1)			
$-38$	(9, 3, 2)	(15, 3, 4)			
$-35$	(2, 2, 2)	(7, 4, 1)			
$-33$	(4, 2, 2)	(8, 1, 3)			
$-31$	(2, 5, 0)	(5, 2, 2)			
$-30$	(3, 5, 0)	(9, 6, 0)			
$-27$	(5, 5, 0)	(8, 4, 1)			
$-26$	(2, 0, 3)	(10, 0, 4)			
$-24$	(4, 0, 3)	(6, 5, 0)			
$-23$	(2, 3, 1)	(7, 2, 2)	(13, 8, 0)		
$-22$	(3, 3, 1)	(5, 0, 3)			
$-19$	(5, 3, 1)	(6, 0, 3)	(7, 5, 0)	(11, 2, 3)	
$-17$	(2, 1, 2)	(10, 3, 2)			
$-16$	(3, 1, 2)	(6, 3, 1)			
$-15$	(2, 4, 0)	(4, 1, 2)	(8, 2, 2)		
$-14$	(3, 4, 0)	(7, 0, 3)	(9, 4, 1)		
$-13$	(4, 4, 0)	(5, 1, 2)			
$-11$	(2, 2, 1)	(5, 4, 0)	(7, 3, 1)	(8, 5, 0)	
$-10$	(3, 2, 1)	(6, 1, 2)	(13, 0, 5)		
$-9$	(4, 2, 1)	(10, 6, 0)			
$-8$	(2, 0, 2)	(6, 4, 0)	(18, 5, 4)		
$-7$	(2, 3, 0)	(3, 0, 2)	(5, 2, 1)	(11, 5, 1)	(14, 7, 1)
$-6$	(3, 3, 0)	(4, 0, 2)	(8, 0, 3)		
$-5$	(2, 1, 1)	(4, 3, 0)	(7, 1, 2)		
$-4$	(3, 1, 1)	(5, 0, 2)	(6, 2, 1)		
$-3$	(2, 2, 0)	(4, 1, 1)	(5, 3, 0)	(7, 4, 0)	(8, 3, 1)
$-2$	(2, 0, 1)	(3, 2, 0)	(9, 2, 2)		
$-1$	(2, 1, 0)	(3, 0, 1)	(4, 2, 0)	(5, 1, 1)	(6, 0, 2)
0	(2, 0, 0)	(3, 1, 0)	(4, 0, 1)	(6, 3, 0)	(12, 4, 2)
1	(3, 0, 0)	(4, 1, 0)	(5, 2, 0)	(7, 2, 1)	(10, 1, 3)
2	(4, 0, 0)	(5, 0, 1)	(6, 1, 1)	(9, 5, 0)	
3	(5, 1, 0)	(8, 1, 2)			
4	(5, 0, 0)	(6, 2, 0)	(7, 0, 2)		
5	(6, 0, 1)	(7, 3, 0)	(8, 4, 0)		
7	(6, 0, 0)	(7, 1, 1)	(9, 0, 3)	(10, 4, 1)	
9	(7, 2, 0)	(8, 2, 1)			
10	(7, 0, 1)	(9, 3, 1)			
12	(7, 0, 0)	(8, 0, 2)			
15	(8, 1, 1)	(16, 2, 5)			
16	(9, 1, 2)	(12, 7, 0)			
17	(8, 2, 0)	(11, 3, 2)	(13, 3, 3)		
18	(8, 0, 1)	(9, 4, 0)			
19	(8, 1, 0)	(10, 2, 2)			
25	(9, 0, 2)	(11, 6, 0)			
28	(9, 1, 1)	(10, 0, 3)			
31	(9, 0, 1)	(10, 3, 1)			
41	(11, 4, 1)	(13, 6, 1)			
53	(10, 1, 0)	(11, 2, 2)	(14, 2, 4)		
71	(11, 1, 2)	(13, 1, 4)			
80	(11, 0, 2)	(12, 6, 0)			
85	(11, 2, 0)	(19, 12, 0)			
89	(13, 4, 2)	(14, 5, 2)			
161	(13, 3, 2)	(14, 3, 3)			
185	(13, 4, 1)	(14, 6, 1)			
215	(13, 1, 2)	(14, 1, 4)	(22, 3, 7)		
578	(15, 5, 0)	(21, 7, 4)			
933	(16, 1, 3)	(20, 3, 6)			
1581	(17, 4, 0)	(20, 6, 4)			
1589	(17, 3, 0)	(19, 5, 4)			
4173	(19, 3, 0)	(20, 5, 4)			
9794	(21, 7, 2)	(27, 8, 6)			
28081	(23, 6, 2)	(26, 7, 6)			
28369	(23, 5, 2)	(25, 6, 6)			
74737	(25, 5, 2)	(26, 6, 6)			
